# Adaptation and Psychometric Evaluation of a Patient Safety Culture Instrument for Home Care—A Multicentre Cross‐Sectional Study

**DOI:** 10.1111/jan.70479

**Published:** 2026-01-09

**Authors:** Tania Martins, Julie Bucher Andary, David Bellagamba, Michael Simon, Franziska Zúñiga

**Affiliations:** ^1^ Nursing Science, Department of Public Health University of Basel Basel Switzerland; ^2^ Association Vaudoise D'aide et de Soins à Domicile Lausanne Switzerland

**Keywords:** home care agencies, safety culture, safety management, surveys and questionnaires, validation study

## Abstract

**Aims:**

To adapt an instrument to measure patient safety culture, as rated by home care workers, and examine its psychometric properties.

**Design:**

A multicentre cross‐sectional psychometric study.

**Methods:**

We adapted the Nursing Home Survey SOPS to measure safety culture in home care. The questionnaire was translated to French following the Translation, Review, Adjudication, Pretest and Documentation (TRAPD) approach. Experts in home care evaluated the content validity of the adapted and translated instrument. To pre‐test the questionnaire, we conducted cognitive interviews. We invited home care workers from two home care agencies in the French‐speaking region of Switzerland to participate in the cross‐sectional study from November to December 2024. We performed confirmatory factor analysis using the R package ‘lavaan’ and assessed convergent, discriminant and known‐groups validity.

**Results:**

Eight experts assessed the content validity of the adapted and translated instrument. Responses from 672 home care workers were analysed. Except for compliance with procedures, all dimensions showed acceptable or good internal consistency. Regarding construct validity, first‐order and second‐order level confirmatory factor analysis showed acceptable model fit. Safety culture correlated with overall patient safety rating and psychosocial safety climate. Regarding known‐groups validity, participants who do not work directly with clients most of the time, and those willing to recommend the organisation rated the safety culture higher.

**Conclusion:**

The psychometric evaluation indicated that the adapted instrument can be used as a valid and targeted tool to assess patient safety climate/culture in Swiss French‐speaking home care agencies.

**Implications for the Profession and/or Patient Care:**

The existence of an adapted and validated instrument for use in home care enables managers to monitor safety culture and develop interventions to improve it and consequently ensure patient safety.

**Impact:**

To the best of our knowledge, there was no instrument specifically targeting the measurement of patient safety culture in the home care setting. The adapted instrument for home care showed to be a valid tool to provide information about safety culture in this setting. The availability of an instrument to measure safety culture in the home care setting can promote its monitoring, raise awareness of safety culture among staff, help managers prioritise key aspects for culture change, and thus improve patient safety. A wider adoption of the same instrument could also facilitate comparative analyses.

**Reporting Method:**

We used the COSMIN guidelines for the psychometric evaluation of the instrument and the STROBE reporting guidelines for the cross‐sectional study.

**Patient or Public Contribution:**

This study did not include patient or public involvement in its design, conduct or reporting.

## What Does This Paper Contribute to the Wider Global Clinical Community?

This research provides a thorough adaptation and psychometric evaluation of a safety culture/climate instrument specifically for the home care setting, addressing its unique characteristics and challenges. The validated instrument supports managers in monitoring safety climate and fostering a positive safety culture within home care agencies.

It enables managers and staff to gain first insights into safety climate and use the results as a starting point to discuss and initiate cultural change.

## 1 Introduction

Unsafe care resulting in unintended patient harm and adverse events accounts for 15% of health spending in Organization for Economic Cooperation and Development (OECD) countries (de Bienassis and Klazinga 2022). With the publication of landmark reports such as ‘To Err is Human’ (Institute of Medicine Committee on Quality of Health Care in America 2000) and more recently with the COVID‐19 pandemic, the awareness of patient safety has become widely acknowledged as an ethical, economic and public health concern driving the need for research and improvement initiatives. Reducing patient harm is considered one of the most effective ways to create value in healthcare, which is best approached using a framework of governance, resilience, culture and transparency (Slawomirski and Klazinga 2022). To maintain safe healthcare systems, both for patients and workers, safety culture has gained increasing importance. Without measuring safety culture in healthcare settings, the ability to identify and reinforce interventions that enhance safety culture and, ultimately, patient safety becomes impossible (de Bienassis and Klazinga 2022). To the best of our knowledge, to date, there is no instrument specifically targeted to measure patient safety culture in home care. Such an instrument could enable the identification of areas for improvement and monitoring of the impact of interventions to promote patient safety in this setting.

### 1.1 Background

The World Health Organization's Global Patient Safety Action Plan 2021–2030 emphasises the importance of embedding a safety culture in healthcare design and delivery. It calls for governments to adopt global approaches to cultivate safety culture, including building competencies for culture change (World Health Organization 2021, 26). The Agency for Healthcare Research and Quality (AHRQ) defines safety culture as the shared values, beliefs and norms among staff within an organisation that influence their safety behaviours (Agency for Healthcare Research and Quality 2022). Nevertheless, different definitions of safety culture exist, as well as of the term safety climate, which is often used interchangeably. However, distinctions have been made between these two concepts. Safety climate is sometimes referred to as the manifestation or expression of safety culture within an organisation, as the latter is invisible (Guldenmund 2000).

Assessing staff perceptions of the safety climate through self‐report questionnaires can be used to assess safety‐related attitudes and perceptions, that is, the surface of the organisation's underlying safety culture, to identify areas for improvement, to track changes over time, to raise awareness of patient safety and for benchmarking purposes (Nieva and Sorra 2003).

Widely used instruments to measure patient safety culture/climate include the Hospital Survey on Patient Safety Culture 2.0 (HSOPSC) SOPS (Agency for Healthcare Research and Quality 2019) and the Safety Attitudes Questionnaire–Short Form (SAQ‐SF), which comprises 60 items and six scales (teamwork climate, job satisfaction, perceptions of management, safety climate, working conditions and stress recognition) (Sexton et al. 2006). Another widely used tool is the Nursing Home Survey on Patient Safety Culture (NHSOPSC) SOPS (Sorra et al. 2008). The NHSOPSC comprises 42 items and 12 composite measures, such as communication openness, handoffs, nonpunitive response to mistakes, staffing and teamwork (Sorra et al. 2008).

In the absence of a targeted instrument specifically designed for the home care setting, the few existing studies in the home care use different instruments, which limits the generalisability and applicability of the collected data for benchmarking and learning purposes (Martins et al. 2025).

Home care refers to a broad range of professional nursing and other healthcare services delivered at the client's home. These services encompass medical treatments and therapies, assistance with personal care and household support. Home care presents distinct safety risks, including care provision in non‐medical environments, healthcare professionals working alone and logistical challenges, such as access to equipment and travel risks (Carpenter et al. 2017). The shift of healthcare to the home leads to an increase in care complexity and the use of medical technologies at home (Ten Haken et al. 2018). The provision of care at home involves collaboration and coordination with sometimes large networks of different professionals and informal caregivers (Renyi et al. 2022), which can increase the complexity of care. In addition, providing care alone in the patient's home, with little opportunity to meet colleagues, poses additional challenges for communication and collaboration within the team. Communication issues are thought to be a cause of adverse events in home care (Masotti et al. 2010).

These differences suggest an adaptation of the instrument that accounts for the specificities of home care. Despite this, the adaptations of the instruments to home care have mainly involved only minor changes in wording (e.g., from ‘patient’ to ‘client’) and the merging or reduction of dimensions (Ree and Wiig 2019; Silverglow et al. 2023). However, given the particularities of the home care setting, a more comprehensive adaptation might be warranted.

The NHSOPSC, although initially designed for nursing homes, has been the more frequently used instrument in the home care setting (Ree 2020; Ree and Wiig 2019, 2020; Viksveen et al. 2022), showing good internal consistency and validity. It has been adapted and validated in different countries, including Switzerland (Zúñiga et al. 2013), France (Teigné et al. 2019), Spain (Vrotsou et al. 2021), Norway (Cappelen et al. 2016) and China (Li et al. 2021). Based on its measurement properties and following a literature search and consultation with 10 experts, we selected the NHSOPSC as the most suitable instrument to be adapted to the home care setting in Switzerland. Although the provision of care in patients' homes poses different risks, as mentioned above, nursing homes and home care agencies share similarities in terms of the types of services provided, as well as population and provider characteristics. The key dimensions of safety culture remain the same, and the NHSOPSC appears best suited to the similar skill and grade mix found in nursing homes and home care.

As the Swiss healthcare system faces significant challenges, including demographic aging, rising costs, workforce shortages, structures overly on acute care and lack of transparency concerning costs and quality of services (Federal Office of Public Health 2019), ensuring a positive safety culture in Swiss home care is essential. In response, the Swiss Federal Office for Public Health, within its quality development strategies, emphasises the importance of measuring and improving safety culture across healthcare institutions (Federal Office of Public Health 2022). In Switzerland, the number of home care clients is projected to increase by 52% by 2040 (Pellegrini et al. 2022). The national policy of fostering home care over residential care leads to increased care complexity, greater use of medical technology at home and the need for support from relatives in caring for clients (Niederhauser and Füglister 2016). Given this growing role of home care, a more thorough adaptation of the NHSOPSC to better target the home care setting could enhance the accuracy and relevance of safety culture measurement in this setting, thereby supporting its monitoring and the development of quality improvement initiatives.

## 2 The Study

### 2.1 Aims

The aims of this study were (1) to adapt, translate and culturally adapt the Nursing Home Survey on Patient Safety Culture (NHSOPSC) SOPS to the home care setting in the French‐speaking region of Switzerland and (2) to examine its psychometric properties.

## 3 Methods

This psychometric evaluation used a stepwise approach to adapt and translate an existing instrument to the home care setting and test it in a multicentre, cross‐sectional study.

**FIGURE 1:**
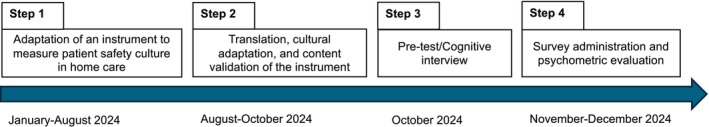
Steps that guided the research process and their respective timeframes.

The steps shown in Figure 1 guided the research process.

### 3.1 Step 1: Adaptation of the Instrument

For the adaptation of the Nursing Home Survey on Patient Safety Culture (NHSOPSC) SOPS (Sorra et al. 2008), we conducted a literature search on patient safety culture and the existing instruments used to measure it. Existing systematic reviews showed similar safety culture dimensions in different instruments (Churruca et al. 2021; Flin et al. 2006; Halligan and Zecevic 2011). In addition, we consulted experts in patient safety culture in Switzerland. We aimed to recruit 10 experts. Expert's eligibility criteria are provided in Table 1. In the first round, experts rated the instrument using a set of predefined criteria (see Table 1) and commented on which aspects specific to this setting needed to be considered. After the first round, we discussed the results with three experts who volunteered to be interviewed. Before the second round of the expert consultations, we adapted the selected instrument based on feedback from the first round, interviews and review of the literature. The selection of additional dimensions and the generation of items were guided by the review of existing instruments, the literature on safety culture and expert consultations. In the second round, experts conducted both dimension‐ and item‐level evaluations using the answer options ‘include as it is’, ‘adapt’ and ‘exclude’, followed by comments to justify their evaluation.

**TABLE 1:** Expert consultation rounds for instrument adaptation.

Expert consultations	Participants	Methods/measurements	Outcome
Round 1: Rating of the instrument	Eligibility criteria: have expertise in the topic of safety culture or alternatively in the field of patient safety (have been engaged in clinical or research work in this field) have knowledge of the Swiss healthcare system be able to understand English.	Online questionnaire assessing instrument's: ease of administration ease of scoring and interpretation appropriateness for benchmark purposes appropriateness to guide the development of interventions dimensions to be included adaptability to the Swiss home care setting weaknesses and strengths of the instrument importance ranking of each assessed aspect (from most important to least important).	Ten experts were recruited.
Interviews	Experts from Round 1 who volunteered to be interviewed.	Online interview with duration of 1 h. Discussion of the results of Round 1 and which aspects specific to home care should be assessed.	Three experts volunteered to an interview.
Round 2: Adaptation of the instrument	Same experts as in Round 1.	Online questionnaire with domain‐level and item‐by‐item evaluation. Answer options: include as it is adapt exclude Comments.	If ≥ 50% of the experts suggested excluding or adapting a dimension or item, it was excluded or adapted after discussion within the study team.

### 3.2 Step 2: Translation, Cultural Adaptation and Content Validation

First, we obtained permission from the original authors of the chosen instrument to adapt and translate it. For the translation and cultural adaptation, we used the TRAPD (Translation, Review, Adjudication, Pretest, Documentation) team approach (Harkness 2008). First, two translators independently translated the entire questionnaire from English to French. One translation was reviewed by a home care worker. Second, the translations were reviewed by the study team (except for one team member), resulting in a single consolidated version of the questionnaire. Third, the study team member who was not involved in the review acted as adjudicator, resolving discrepancies and deciding whether the questionnaire was ready for pretesting. Fourth, we assessed its content validity, that is, the extent to which the instrument adequately measures safety culture/climate. We aimed to recruit 10 experts (see Table 2 for the eligibility criteria). The experts rated independently the relevance of each item on a 4‐point scale from 1 = ‘not relevant’ to 4 = ‘highly relevant’. The item‐level content validity index (I‐CVI) was calculated as the proportion of experts rating an item as either 3 or 4. Items with I‐CVI < 0.78 (Polit et al. 2007) were reviewed or removed. The I‐CVI values are provided in Appendix S1. In addition, experts evaluated the comprehensibility and comprehensiveness of the items and dimensions. Finally, the pre‐test was conducted through cognitive interviews with home care workers (see Step 3).

**TABLE 2:** Steps in the translation, cultural adaptation and content validation.

Purpose	Participants	Methods/measurements	Outcome
Translation and cultural adaptation of the adapted instrument	Translators, study team and one home care worker.	TRAPD (Translation, Review, Adjudication, Pretest, Documentation) team approach (Harkness 2008). Two independent translations English‐French Review by the research group Adjudication by a study member Pre‐test (see Step 3)	A pre‐final, translated and adapted French version of the instrument was developed.
Assessment of the content validity	Purposive sample of 10 experts. Eligibility criteria: at least 5 years of professional experience in home healthcare (clinical, research or education) work or have worked in home healthcare in the French‐speaking region of Switzerland and very high proficiency in French.	Online questionnaire. Experts rated and commented on: relevance (from 1 = ‘not relevant’ to 4 = ‘highly relevant’) comprehensibility (yes/no) comprehensiveness (are items or dimensions missing) (yes/no) overall content validity of items and dimensions We collected socio‐demographic characteristics of the experts.	Items with low ratings (I‐CVI < 0.78) (Polit et al. 2007) on the relevance, items/dimensions rated as not comprehensible or not comprehensive were reviewed or removed.

*Note:* I‐CVI, item content validity index.

### 3.3 Step 3: Pre‐Test/Cognitive Interview

After adapting the questionnaire based on the content validation, we pre‐tested the instrument with a convenience sample of home care workers from the target population to assess the feasibility and clarity of the instrument. Eligible for participation were (a) all home care workers, such as registered nurses, nurse assistants, administrative personnel and home care aides; (b) those working in the home care agency for at least 3 months; and (c) those who were 18 years or older. Eligible home care workers were invited by the home care managers by email, which included an explanation of the study and the aims of the cognitive interview. We aimed to interview 10 home care workers. Participants who volunteered to participate received both written and verbal information about the purpose of the study and the procedure for the interview. Written informed consent was obtained prior to the interview. The study team conducted online cognitive interviews using the think‐aloud method (Ericsson and Simon 1980), during which we assessed, for example, participants' ability to recall information, their confidence in the responses and the reasoning behind their answers. We also assessed the feasibility of the survey, including the length of the questionnaire, clarity of instructions and adequacy of the font size. Each interview had a duration of approximately 1 h. Unclear items were revised for clarity, reworded, or reformatted as needed, and then re‐tested in the final cognitive interviews.

### 3.4 Step 4: Survey Administration and Psychometric Evaluation

#### 3.4.1 Design, Setting and Sampling

We conducted a multicentre, cross‐sectional study in a convenience sample of two home care agencies in the French‐speaking region of Switzerland. Eligible for inclusion were (a) all home care workers from any profession aged 18 years or older, (b) employed by the home care agency for at least 3 months and (c) able to understand written French. We excluded participants who did not answer at least 80% of the questionnaire. For sample size estimation, we used a respondent‐to‐item ratio of 10:1, suggesting a total of at least 560 respondents (Nunnally 1978).

#### 3.4.2 Data Collection

An email with the link to the online questionnaire (using REDCap electronic data capture tools) (Harris et al. 2019) was sent to all eligible participants by the home care managers. Data were collected between November and December 2024. Home care managers received weekly response rates and reminded all invited home care workers (by email, intranet news and in meetings) at 2 and 4 weeks.

#### 3.4.3 Variables and Measurements

##### 3.4.3.1 Safety Culture

Based on the former steps, an adaptation of the NHSOPSC was used. The original NHSOPSC instrument has 42 items on 12 dimensions (Sorra et al. 2008). Items are scored on a 5‐point Likert scale ranging from 1 = ‘Strongly disagree’ or ‘Never’ to 5 = ‘Strongly agree’ or ‘Always’ and with the answer option ‘Does not apply or don't know’. The average percent positive scores are then calculated for each dimension. For positively formulated items, the percentage of respondents answering ‘Strongly agree’, ‘Agree’/‘Always’, ‘Most of the time’ are considered positive. For negatively formulated items, ‘Strongly disagree’/‘Disagree’, ‘Never’/‘Rarely’ are considered positive. In the literature, authors often calculate mean scores instead of the percentage of positive responses.

##### 3.4.3.2 Overall Patient Safety Ratings

We used a single‐item adapted from the NHSOPSC (Sorra et al. 2008) assessing overall patient safety rating (adapted item: *Please give this home care organisation an overall rating on client safety*) to evaluate convergent validity.

A single item on willingness to recommend as a safe home care organisation (adapted item: *I would tell friends that this is a safe home care organisation for their family*) with answer options ‘Yes’, ‘Maybe’ and ‘No’ was used to evaluate known‐groups validity.

##### 3.4.3.3 Psychosocial Safety Climate

We used the 12‐item validated Psychosocial Safety Climate‐12 (PSC‐12) instrument to assess four dimensions of psychosocial safety climate (Hall et al. 2010). Answer options ranged from 1 = ‘Strongly disagree’ to 5 = ‘Strongly agree’. Both the total PSC‐12 sum score and the sum scores for each dimension can be used. To assess convergent validity, we used the total PSC‐12 sum score. While no prior psychometric evaluation exists for the French‐speaking region of Switzerland, the PSC‐12 has demonstrated good validity and reliability among French‐speaking participants (Huyghebaert et al. 2018; Truchon et al. 2025). Studies conducted in other linguistic and cultural contexts (Abu Bakar et al. 2025; Inoue et al. 2021) have also reported good psychometric properties, further supporting the instrument's validity and reliability. In our sample, the Cronbach's alpha of the instrument's dimensions ranged between 0.85 and 0.91. Both psychosocial safety climate and patient safety climate reflect safety‐oriented organisational climates—manifested in artefacts, espoused values and underlying assumptions about safety—within the broader construct of organisational culture (Guldenmund 2000; Schein and Schein 2016). Management commitment to safety is a central and common driver of both climates, shaping safety priorities and the overall safety climate of an organisation and influencing patients and workers' safety (Flin 2007).

##### 3.4.3.4 Socio‐Demographic Characteristics

Age, gender, professional category, work years in the current home care organisation, percentage of employment and involvement in the provision of direct client care most of the time were assessed to describe the study participants.

#### 3.4.4 Data Analysis

Descriptive statistics, such as frequencies, means and their standard deviations, or medians and their interquartile ranges, were presented to describe item responses and respondents' socio‐demographic characteristics. Negatively formulated items of the safety culture instrument were reverse coded, so that for all items higher scores indicate a more positive patient safety climate.

Data analysis was performed using R version 4.2.3 (R Core Team 2023) and the R packages ‘psych’ 2.4.12 (Revelle 2024) and ‘lavaan’ 0.6‐19 (Rosseel 2012).

##### 3.4.4.1 Response Variability

The percentage of missing responses, ‘Does not apply or don't know’, floor and ceiling effects were examined for each item. The percentage of participants choosing the highest or lowest answer option was calculated to evaluate ceiling and floor effects. Commonly, ≤ 15% of floor or ceiling effects are considered to meet the standards (for health status surveys) (McHorney and Tarlov 1995). We used the Kruskal–Wallis test to examine whether the number of missing responses and ‘Does not apply or don't know’ answers differed significantly (*p* value < 0.05) based on respondents' socio‐demographic characteristics.

If an item had more than 30% missing responses or ‘Does not apply or don't know’ answers, it was considered for removal based on both the extent of missing data and the psychometric evaluation. We considered response variability low in items where 90% or more chose the answers ‘Agree’ or ‘Strongly agree’/‘Most of the times’ or ‘Always’.

##### 3.4.4.2 Internal Consistency

For internal consistency, we estimated Cronbach's alpha and composite reliability (CR) for each of the safety culture dimensions. Values of at least 0.70 were considered acceptable, ≥ 0.80 as good and ≥ 0.90 as excellent reliability (Nunnally 1978). However, values above 0.90 may indicate redundancy between items (Streiner 2003). Inter‐item and item‐total correlations were examined to assess the homogeneity and to control for overlap of the newly developed items using Spearman correlation coefficients (*r*_s_).

##### 3.4.4.3 Construct Validity

As we aimed to test the fit of the data to the a priori defined safety culture dimensions (from the NHSOPSC survey (Sorra et al. 2008), we conducted confirmatory factor analysis (CFA)). To assess the construct validity of the patient safety culture instrument, we performed individual level one‐factor (for each dimension) CFA models for ordinal data using weighted least square mean and variance adjusted estimator. Missing data, including the ‘Does not apply or don't know’ response options, were handled through pairwise deletion.

Measurement model fit statistics were computed for dimensions with four or more items. For the dimensions with three or fewer items, only factor loadings were assessed. Although the AHRQ does not explicitly mention that an overall safety culture score should be computed, as many authors compute an overall safety culture score, we also performed a second‐order level CFA. We fitted the second‐order CFA model, including all safety culture dimensions, allowing them to correlate with each other. We considered as reasonably good fit goodness‐of‐fit indexes as follows: comparative fit index (CFI) and Tucker‐Lewis Index (TLI) values close to 0.95 or greater (between 0.90 and 0.95 is indicative of acceptable model fit), root mean square error of approximation (RMSEA) values close to 0.06 or below 0.05, and the standardised root mean square residual (SRMR) close to 0.08 or below (Hu and Bentler 1999). In addition, we reported chi‐square statistics (*χ* ^2^) along with the degrees of freedom (df).

##### 3.4.4.4 Convergent and Discriminant Validity

We analysed convergent validity of the constructs by calculating the average variance extracted (AVE). For adequate convergent validity the AVE value should be ≥ 0.50, that is, at least 50% of the variance on the observed variable should be explained by the latent construct (Fornell and Larcker 1981). We also correlated (Spearman *r*_s_) the dimensions survey scores (and total survey scores) with the single‐item on overall patient safety rating, the one on willingness to recommend and with psychosocial safety climate scale (PSC‐12) (Hall et al. 2010). Previous studies revealed a moderate to strong relationship between safety culture and overall patient safety rating (Cappelen et al. 2016; Silverglow et al. 2023) and willingness to recommend (Cappelen et al. 2016). We hypothesised a moderate (*r*_s_ ≥ 0.35–0.50) or strong (*r*_s_ > 0.50) positive relationship between safety culture, overall patient safety ratings, willingness to recommend and psychosocial safety climate.

We assessed discriminant validity through the Heterotrait‐Monotrait ratio (HTMT) of correlations. HTMT ratios between the safety culture dimensions below 0.85 support the distinctiveness of the different dimensions (Henseler et al. 2015).

##### 3.4.4.5 Internal and Known‐Groups Validity

For internal validity we determined the intercorrelations between the safety culture dimensions (*r*_s_ = 0.30–0.70). For known‐groups validity, differences in safety culture scores were analysed between those who were most of the time involved in direct client care and those who were not. Based on a previous study (Vrotsou et al. 2021), we expected those not working directly with clients to rate safety culture higher. In addition, we expected those who reported they would tell friends that the home care organisation is a safe place for their families to rate safety culture higher.

### 3.5 Ethical Considerations

The Ethics Committee of canton Vaud ruled that the study was exempt from the Swiss human‐research act (Req‐2024‐00123). Data confidentiality and anonymity were ensured throughout the research process. The first page of all questionnaires informed participants about the purpose of the study, how their data would be handled, the confidentiality of their responses, and that their participation was voluntary. Participants were asked to opt in to participation on the first page. If they did not agree to participate, the survey ended automatically.

## 4 Results

### 4.1 Step 1: Adaptation of the Instrument

Ten experts in safety culture or patient safety participated in the adaptation of the instrument: three psychologists, three nurses, two medical doctors, one pharmacist and one healthcare manager. Based on a review of the literature, the results of expert consultations and interviews, we adapted the instrument. For example, we added the reporting of client safety events dimension, as this was mentioned by 90% (*n* = 9) of the experts as a dimension that should be included. This dimension, already included in the Hospital Survey 2.0 SOPS, is defined as the extent to which mistakes of the following types are reported: (1) mistakes caught and corrected before reaching the client and (2) mistakes that could have harmed the client but did not. We retained the original definition, modifying only the term ‘patient’ to ‘client’. In addition to the new dimension, we added new items to address topics such as coordination within the organisation and with external healthcare/service providers; loss of information due to clients seeing different staff members; provision of specific training on client safety and adequate resources; integration of lessons learned from errors; and provision of support for staff involved in safety errors (see Appendix S2).

### 4.2 Step 2: Translation and Content Validation

Nine experts, including seven nurses, one economist and one healthcare quality engineer, assessed the content validity of the adapted and translated instrument. All had training or roles in quality and safety in home care, with more than 5 years of experience in home care. The Item Content Validity Index (I‐CVI) values ranged from 0.50 to 1.00. The Scale Content Validity Index (S‐CVI) ranged between 0.66 and 0.97. Seven items had a low I‐CVI (< 0.78), of which six were reviewed and one was suppressed. We added definitions (as clickable info buttons) for the terms that were considered difficult to understand or unclear, such as harm (*préjudice*), turnover (*turnover*) and client safety (*sécurité des clients*).

### 4.3 Step 3: Pre‐Test/Cognitive Interviews

Eight home care workers participated in the pre‐test/cognitive interviews: six registered nurses and two nurse assistants. For the two remaining home care workers, cognitive interviews could not be conducted within the time frame due to scheduling constraints. Minor wording changes were made to improve the clarity of the items. The English version of the administered questionnaire can be found in Appendix S3.

### 4.4 Step 4: Psychometric Evaluation

Of the 1609 home care workers to whom the link to the final online survey was sent, 674 completed the questionnaire (response rate: 42.0%). Two questionnaires were excluded, one because less than 80% of the questionnaire was completed and one because the participant answered ‘Does not apply or don't know’ to all safety culture survey items, resulting in 672 questionnaires for analysis.

Most of the participants were female (82.5%) and had direct interaction with clients most of the time (85.1%) (see Table 3).

**TABLE 3:** Socio‐demographic characteristics of study participants (*n* = 672).

Characteristics	*n* (%)	Missing (%)
Gender		5 (0.7%)
Female	550 (82.5%)	
Male	104 (15.6%)	
Other	13 (1.9%)	
Age		23 (3.4%)
18–27	49 (7.5%)	
28–37	173 (26.7%)	
38–47	159 (24.5%)	
48–57	189 (29.1%)	
58–64	79 (12.2%)	
Professional category		3 (0.4%)
Registered nurses, clinical experts and care coordinators	276 (41.3%)	
Assistants, students and support staff	209 (31.3%)	
Other allied health professionals	53 (7.9%)	
Administration and technical staff	49 (7.3%)	
Managers	41 (6.1%)	
Community health agents and nursing assistants	35 (5.2%)	
Other	6 (0.9%)	
Years in the current organisation		17 (2.5%)
< 1	98 (14.9%)	
1–5	288 (44.1%)	
6–10	133 (20.3%)	
11–15	73 (11.1%)	
16–20	26 (4.0%)	
≥ 21	37 (5.6%)	
Percentage of employment		23 (3.4%)
≤ 50%	74 (11.4%)	
51%–80%	435 (67.1%)	
81%–100%	140 (21.5%)	
Direct interaction with clients most of the time		9 (1.3%)
Yes	564 (85.1%)	
No	99 (14.9%)	

#### 4.4.1 Response Variability

Of the 672 participants, 70.5% (*n* = 474) completed the questionnaire without missing data. Missing values per item (of the safety culture instrument) varied between 0% and 2.2% (item R6: *In this organisation, staff are blamed when a client is harmed*). No significant differences in the number of missing values were found between participants with different socio‐demographic characteristics.

One item, item B5 (*This organisation is doing more for client safety now than it did one year ago*), had a percentage of ‘Does not apply or don't know’ responses above 30% (33.5%). Significant differences in the ‘Does not apply or don't know’ answers were found based on respondents' work experience in the current organisation (*p* < 0.001) (higher in < 1 year), involvement in direct client care (*p* < 0.001) (higher in those who reported no direct client care), professional category (*p* < 0.001) (higher in administration and technical staff) and percentage of employment (*p* < 0.01) (higher in respondents with an employment percentage ≤ 50%).

The percentage of positive responses, ‘Does not apply or don't know’ and missing responses, floor and ceiling effects, as well as means and their standard deviations, are provided in Table 4. Within each item, the percentage of positive responses, including missing responses and ‘Does not apply or don't know’ answers were for all items less than 90%, except for item T1: *Staff in this organisation treat each other with respect* with 90% of positive responses (i.e., both ‘Agree’ and ‘Strongly agree’), showing low variability. Floor effects were < 15% for all items, whereas ceiling effects exceeded 15% for some items.

**TABLE 4:** Descriptive statistics of the obtained responses.

Dimensions/items	Mean (SD)	Does not apply or don't know, *n* (%)	Missing responses, *n* (%)	Floor effects^a^, *n* (%)	Ceiling effects^b^, *n* (%)
Teamwork					
T1. In this organisation, staff treat each other with respect.	4.4 (0.7)	0	1 (0.1)	4 (0.6)	358 (53.3)
T2. In this organisation, staff support one another.	4.2 (0.8)	4 (0.6)	1 (0.1)	6 (0.9)	280 (41.7)
T3. In this organisation, staff feel they are part of a team.	4.2 (0.7)	7 (1.0)	7 (1.0)	1 (0.1)	275 (40.9)
T4. In this organisation, when someone gets really busy, the other team members are ready to help out (e.g., by taking over a visit, a service or other duties).	4.0 (0.9)	35 (5.2)	6 (0.9)	11 (1.6)	192 (28.6)
T5. In this organisation, staff work together as a well‐coordinated team.	4.2 (0.8)	4 (0.6)	5 (0.7)	2 (0.3)	257 (38.2)
Staffing					
S1. In this organisation, there is enough staff to handle the workload.	3.2 (1.1)	19 (2.8)	0	42 (6.2)	98 (14.5)
S2. In this organisation, staff generally have to hurry because they have too much work to do. (*negatively worded*)	2.8 (1.1)	23 (3.4)	2 (0.3)	83 (12.3)	41 (6.1)
S3. In this organisation, staff have enough time to respond to clients' needs.	3.5 (1.0)	29 (4.3)	3 (0.4)	14 (2.1)	98 (14.6)
S4. In this organisation, it's hard to keep clients safe because of a high turnover rate. (*negatively worded*)	3.1 (1.1)	71 (10.6)	7 (1.0)	49 (7.3)	53 (7.9)
Compliance with procedures					
C1. In this organisation, staff follow the procedures in place (e.g., procedure in the event of a fall, death, etc.) to care for clients.	4.3 (0.7)	41 (6.1)	4 (0.6)	0 (0)	246 (36.6)
C2. In this organisation, to get the work done easier, staff often ignore the procedures in place in the organisation. (*negatively worded*)	3.6 (1.0)	85 (12.6)	6 (0.9)	23 (3.4)	92 (13.7)
Training and skills					
Z1. In this organisation, staff get the training they need to feel confident in the performance of their job.	4.0 (0.9)	8 (1.2)	2 (0.3)	5 (0.7)	211 (31.4)
Z2. In this organisation, staff have enough training on how to handle difficult situations with the clients.	3.6 (0.9)	35 (5.2)	6 (0.9)	9 (1.3)	100 (14.9)
Z3. In this organisation, staff get specific training in client safety.	3.8 (0.9)	50 (7.4)	3 (0.4)	5 (0.7)	138 (20.5)
Nonpunitive response to mistakes					
R1. In this organisation, when staff makes errors, the organisation focuses on learning rather than blaming individuals.	3.8 (1.0)	66 (9.8)	2 (0.3)	17 (2.5)	135 (20.1)
R2. In this organisation, staff are afraid to report their mistakes. (*negatively worded*)	2.6 (1.0)	85 (12.6)	5 (0.7)	23 (3.4)	71 (10.6)
R3. In this organisation, staff are treated fairly when they make mistakes.	3.7 (1.0)	117 (17.4)	3 (0.4)	12 (1.8)	110 (16.4)
R4. In this organisation, staff feel safe reporting their mistakes.	3.8 (0.9)	51 (7.6)	1 (0.1)	6 (0.9)	121 (18.0)
R5. In this organisation, staff involved in client safety errors do not receive sufficient support. (*negatively worded*)	3.5 (0.9)	151 (22.5)	0	12 (1.8)	51 (7.6)
R6.^c^ In this organisation, staff are blamed when a client is harmed. (*negatively worded*)	3.5 (1.1)	119 (17.7)	15 (2.2)	19 (2.8)	101 (15.0)
Handoffs and information exchange					
H1. Staff have all the information they need to know before taking care of a new client for the first time.	3.9 (0.8)	35 (5.2)	1 (0.1)	2 (0.3)	120 (17.9)
H2. Staff are notified when there is a change in a client's care plan/service provision before the visit.	3.4 (1.0)	48 (7.1)	5 (0.7)	26 (3.9)	113 (16.9)
H3. Staff have all the information they need when clients are transferred from other healthcare facilities (hospitals, nursing homes, etc.).	3.6 (0.9)	55 (8.2)	2 (0.3)	3 (0.4)	87 (13.0)
H4. Staff have all the information they need to care for clients.	4.1 (0.6)	27 (4.0)	2 (0.3)	1 (0.1)	172 (25.6)
H5. Staff have all the information they need after clients had a medical consultation (general practitioner, specialist, etc.).	3.5 (1.0)	79 (11.8)	2 (0.3)	11 (1.6)	75 (11.2)
H6. Staff have all the information they need when clients are transferred from one team to another within the organisation.	4.0 (0.8)	99 (14.7)	1 (0.1)	2 (0.3)	160 (23.8)
H7.^c^ In this organisation, important information is often lost because clients see different staff.	3.3 (0.9)	76 (11.3)	3 (0.4)	13 (1.9)	57 (8.5)
Feedback and communication about incidents					
F1. In this organisation, when staff report something that could harm a client to their manager, someone in the organisation takes care of it.	4.4 (0.7)	62 (9.2)	2 (0.3)	1 (0.1)	309 (46.0)
F2. In this organisation, we reflect together about ways to keep incidents from happening again.	4.2 (0.9)	49 (7.3)	4 (0.6)	6 (0.9)	256 (38.1)
F3. In this organisation, staff tell their manager if they see something that might harm a client.	4.4 (0.6)	37 (5.5)	2 (0.3)	0 (0)	311 (46.3)
F4. In this organisation, staff discuss ways to ensure clients' safety.	4.3 (0.7)	42 (6.2)	7 (1.0)	1 (0.1)	293 (43.6)
F5. In this organisation, staff are informed about corrective measures or improvements made based on incident reports.	4.1 (0.9)	66 (9.8)	0	6 (0.9)	226 (33.6)
Communication openness					
O1. In this organisation, staff ideas and suggestions are valued.	4.0 (0.8)	30 (4.5)	2 (0.3)	7 (1.0)	197 (29.3)
O2. In this organisation, it is easy for staff to speak up about problems.	4.0 (0.9)	31 (4.6)	1 (0.1)	6 (0.9)	205 (30.5)
O3. In this organisation, communication is founded on trust.	4.4 (0.8)	19 (2.8)	5 (0.7)	6 (0.9)	337 (50.1)
O4.^c^ In this organisation, staff are afraid to ask questions when something does not seem right. (*negatively worded*)	3.8 (1.0)	66 (9.8)	2 (0.3)	23 (3.4)	159 (23.7)
Supervisor or manager expectations and actions promoting client safety					
P1. My supervisor or manager listens to staff ideas and suggestions about client safety.	4.5 (0.6)	31 (4.6)	1 (0.1)	1 (0.1)	380 (56.5)
P2. My supervisor or manager expresses satisfaction when work is carried out in compliance with safety procedures.	4.3 (0.8)	35 (5.2)	1 (0.1)	6 (0.9)	335 (49.8)
P3. My supervisor or manager takes action to address client safety concerns that are brought to their attention.	4.5 (0.7)	33 (4.9)	0	1 (0.1)	361 (53.7)
P4. My supervisor or manager is actively committed to client safety.	4.5 (0.7)	34 (5.1)	5 (0.7)	2 (0.3)	360 (53.6)
Overall perceptions of client safety					
B1. Clients are well cared for in this organisation.	4.3 (0.7)	17 (2.5)	3 (0.4)	1 (0.1)	294 (43.7)
B2. This organisation does a good job to ensure clients safety.	4.2 (0.8)	32 (4.8)	1 (0.1)	1 (0.1)	234 (34.8)
B3. This organisation provides safe care/services to clients.	4.3 (0.7)	20 (3.0)	3 (0.4)	2 (0.3)	269 (40.0)
B4. The safety of care/services is not neglected in favour of greater productivity.	3.7 (1.1)	63 (9.4)	3 (0.4)	23 (3.4)	15 (23.1)
B5.^c^ This organisation is doing more for client safety now than it did 1 year ago.	3.3 (1.0)	225 (33.5)	3 (0.4)	19 (2.8)	66 (9.8)
Management support for client safety					
M1. The direction listens to staff ideas and suggestions to improve client safety.	4.0 (0.9)	46 (6.8)	10 (1.5)	8 (1.2)	210 (31.2)
M2. The actions of the management show that client safety is one of the top priorities.	4.0 (0.9)	42 (6.2)	3 (0.4)	4 (0.6)	204 (30.4)
M3. The direction seems interested in client safety only after an adverse event happens. (*negatively worded*)	3.7 (1.1)	57 (8.5)	4 (0.6)	26 (3.9)	161 (24.0)
M4. The direction provides adequate resources (e.g., materials, technological resources, human resources, etc.) to improve client safety.	3.9 (0.9)	33 (4.9)	3 (0.4)	9 (1.3)	171 (25.4)
M5. The direction asks staff how the organisation can improve client safety.	3.7 (1.0)	73 (10.9)	2 (0.3)	17 (2.5)	145 (21.6)
Organisational learning					
L1. In this organisation, the same mistakes happen again and again. (*negatively worded*)	3.8 (1.0)	66 (9.8)	0	18 (2.7)	153 (22.8)
L2. In this organisation, it is easy to make changes to improve client safety.	3.7 (0.9)	54 (8.0)	2 (0.3)	6 (0.9)	118 (17.6)
L3. This organisation is always doing things to improve client safety.	4.0 (0.8)	36 (5.4)	2 (0.3)	3 (0.4)	197 (29.3)
L4. When this organisation makes changes to improve client safety, it checks to see if the changes worked.	3.9 (0.9)	107 (15.9)	3 (0.4)	5 (0.7)	135 (20.1)
Reporting client safety events					
A1. When a mistake is caught and corrected before reaching the client, it is reported …	4.0 (1.0)	150 (22.3)	1 (0.1)	5 (0.7)	166 (24.7)
A2. When a mistake reaches the client, but did not harm him, it is reported …	3.8 (1.0)	159 (23.7)	8 (1.2)	9 (1.3)	145 (21.6)

*Note:* Negatively worded items were reverse coded for the analysis. Answer options: 1 = ‘Strongly disagree’ to 5 = ‘Strongly agree’, 1 = ‘Never’ to 5 = ‘Always’, 10 = ‘Does not apply or don't know’. This English version of the items is an unofficial translation.

Abbreviation: SD, standard deviation.

^a^ Answer option 1.

^b^ Answer option 5.

^c^ These items (R6, H7, O4, B5) were later removed from the instrument.

#### 4.4.2 Internal Consistency

Cronbach's alpha of the safety culture dimensions ranged from 0.39 (compliance with procedures) to 0.89 (reporting client safety events, supervisor and manager expectation and actions promoting client safety). Except for the dimension compliance with procedures, all dimensions had Cronbach's alpha above 0.70 (see Table 5). The handoffs and information exchange dimension showed higher internal consistency without item H7. As this item had also high ‘Does not apply or don't know’ answers, it was removed.

**TABLE 5:** Factor loadings, internal consistency, composite reliability and average variance extracted.

Dimensions	Items	Second‐order model factor loadings (standardised)	Cronbach's alpha (*α*) (95% CI), CR and AVE
Teamwork	T1	0.755	*α* = 0.82 (95% CI 0.79–0.84) CR = 0.83 AVE = 0.60
	T2	0.765	
	T3	0.841	
	T4	0.558	
	T5	0.926	
Staffing	S1	0.706	*α* = 0.78 (95% CI 0.74–0.81) CR = 0.78 AVE = 0.53
	S2	0.555	
	S3	0.819	
	S4	0.817	
Compliance with procedures	C1	0.713	*α* = 0.39 (95% CI 0.28–0.49) CR = 0.51 AVE = 0.56
	C2	0.494	
Training and skills	Z1	0.812	*α* = 0.70 (95% CI 0.64–0.75) CR = 0.71 AVE = 0.52
	Z2	0.590	
	Z3	0.730	
Nonpunitive response to mistakes	R1	0.542	*α* = 0.76 (95% CI 0.72–0.81) CR = 0.79 AVE = 0.48
	R2	0.566	
	R3	0.756	
	R4	0.825	
	R5	0.709	
Handoffs and information exchange	H1	0.805	*α* = 0.85 (95% CI 0.83–0.87) CR = 0.86 AVE = 0.60
	H2	0.656	
	H3	0.784	
	H4	0.852	
	H5	0.778	
	H6	0.788	
Feedback and communication about incidents	F1	0.827	*α* = 0.82 (95% CI 0.79–0.84) CR = 0.84 AVE = 0.60
	F2	0.860	
	F3	0.632	
	F4	0.775	
	F5	0.765	
Communication openness	O1	0.818	*α* = 0.75 (95% CI 0.70–0.79) CR = 0.77 AVE = 0.62
	O2	0.670	
	O3	0.875	
Supervisor and manager expectations and actions promoting client safety	P1	0.890	*α* = 0.89 (95% CI 0.87–0.91) CR = 0.90 AVE = 0.81
	P2	0.857	
	P3	0.915	
	P4	0.959	
Overall perceptions of client safety	B1	0.844	*α* = 0.78 (0.74–0.82) CR = 0.85 AVE = 0.78
	B2	0.956	
	B3	0.838	
	B4	0.642	
Management support for client safety	M1	0.878	*α* = 0.86 (95% CI 0.84–0.88) CR = 0.87 AVE = 0.65
	M2	0.874	
	M3	0.658	
	M4	0.811	
	M5	0.825	
Organisational learning	L1	0.768	*α* = 0.84 (95% CI 0.81–0.87) CR = 0.84 AVE = 0.65
	L2	0.771	
	L3	0.906	
	L4	0.797	
Reporting client safety events	A1	0.946	*α* = 0.89 (95% CI 0.87–0.92) CR = 0.91 AVE = 0.89
	A2	0.935	

Abbreviations: *α*, Cronbach's alpha; AVE, average variance extracted (should be ≥ 0.50); CI, confidence interval; CR, composite reliability (omega) (should be > 0.70).

Item R6 had an item‐total and within its dimension Spearman correlation (*r*_s_) value of less than 0.30. In addition, most of the inter‐item correlations (*r*_s_) for item R6 were not statistically significant. Item B5 also showed mostly non‐significant inter‐item correlations. Some inter‐item correlations with items R6 and B5 were also negative, which was not the case for all other items. Item‐total *r*_s_ values for all other items ranged from 0.33 to 0.75 (see Appendix S4). The dimensions of nonpunitive response to errors, overall perception of client safety and communication openness showed better internal consistency without the items R6, B5 and O4, respectively. However, these three items were initially included in the CFA for testing, but they showed low factor loadings and resulted in a poorer model fit; their removal was therefore confirmed.

#### 4.4.3 Construct Validity

The individual level one‐factor CFA models for dimensions with more than three items showed acceptable goodness‐of‐fit indexes (see Appendix S5). However, the RMSEA were for some dimensions over 0.08. The second‐order CFA model including all dimensions and allowing them to correlate with each other showed standardised factor loadings above 0.50 and statistically significant (*p* < 0.001), except for the item C2 of the compliance with procedures dimension, which had factor loadings below 0.50 (see Table 5).

The model goodness‐of‐fit indexes for the 13‐factor model showed adequate fit to the data; *χ* ^2^ (3802)/df (1261) = 3.0, *p* value = 0.000, CFI = 0.943, TLI = 0.940, RMSEA = 0.055 (90% CI 0.053–0.057), SRMR = 0.060. The French version of the instrument can be found in https://doi.org/10.5281/zenodo.15421522.

#### 4.4.4 Convergent and Discriminant Validity

The average variance extracted (AVE) for all dimensions, except for the dimension nonpunitive response to errors (0.48), was above 0.50 (see Table 5).

The dimensions survey scores showed moderate or strong correlation (Spearman *r*_s_), which reached statistical significance (*p* < 0.05) with overall client safety rating and psychosocial safety climate (see Table 6).

**TABLE 6:** Results of convergent validity.

Dimensions	Overall client safety rating (Spearman correlation *r*_s_)	Psychosocial safety climate (PSC‐12) (Spearman correlation *r*_s_)
Teamwork	0.47	0.53
Staffing	0.50	0.52
Compliance with procedures	0.37	0.27
Training and skills	0.50	0.43
Nonpunitive response to mistakes	0.51	0.58
Handoffs and information exchange	0.55	0.47
Feedback and communication about incidents	0.59	0.55
Communication openness	0.52	0.61
Supervisor or manager expectations and actions promoting client safety	0.52	0.59
Overall perceptions of client safety	0.68	0.61
Management support for client safety	0.66	0.61
Organisational learning	0.66	0.62
Reporting client safety events	0.46	0.39
Total/Overall safety culture mean score	0.72	0.68

*Note:* To assess correlations between dimensions and items, we used the mean scores of the safety culture dimensions.

The HTMT ratios between the safety culture dimensions were all below the recommended threshold of 0.85 (Henseler et al. 2015), with the exception of the ratios between the dimensions organisational learning and management support for client safety (0.92), organisational learning and overall perceptions of client safety (0.91) and overall perceptions of client safety and management support for client safety (0.87), which indicates that these three dimensions might not be totally distinct from each other, that is, that there might be some overlap between these dimensions.

#### 4.4.5 Internal and Known‐Groups Validity

For internal validity, we determined the intercorrelations between the safety culture dimensions. All intercorrelations were within the range of 0.30–0.70 except for the correlations between supervisor or manager expectations and actions promoting client safety and compliance with procedures (0.27), overall perceptions of client safety and both management support for client safety (0.73) and organisational learning (0.75), and management support for client safety and organisational learning (0.79) (see Appendix S6). We tested a model with the three dimensions of overall perceptions of client safety, management support for client safety and organisational learning, combined into one, but as the model fit did not improve significantly, we kept the dimensions separate.

For known‐groups validity, we expected those not involved in direct client care most of the time to rate safety culture higher. In terms of the total mean score, respondents with no involvement in direct client care had a 0.06 higher mean score (3.98) than the ones involved in direct client care (3.92); however, this difference was not statistically significant (*p* = 0.257). Respondents who answered ‘Yes’ to the item *I would tell friends that this is a safe home care organisation for their family* had a statistically significant (*p* < 0.001) higher overall mean safety culture score than respondents who answered ‘Maybe’ or ‘No’.

## 5 Discussion

The goal of the study was to adapt the NHSOPSC SOPS (Sorra et al. 2008) to the home care setting and examine its psychometric properties. Overall, the adapted instrument showed acceptable psychometric properties and can thus serve as a reliable instrument to investigate patient safety culture in Swiss French‐speaking home care agencies.

Through a literature search and expert consultations, we adapted the NHSOPSC (Sorra et al. 2008) instrument to the Swiss home care setting. Unlike previous adaptations of instruments to the home care setting, which consisted of minor wording changes, merging or reducing dimensions, we aimed to assess the appropriateness of the items to the home care setting and to modify the instrument to reflect the specificities of home care. This resulted in adaptations, removals and addition of items. For example, we adapted items relating to *staff being told* to *staff having*, for example, the information needed, as staff are not told what they need to know before taking care of clients; rather, the information is available or not in the electronic health record. In addition, we removed, for example, the item related to *shift changes* and the item related to *management often walks around*, as these items were considered irrelevant for the home care setting. We added items, for instance, to address the coordination and handoffs within the team, to assess the exchange of information with outpatient and inpatient care/service providers, and to assess the availability of adequate resources to improve client safety, as these are important challenges in home care. We believe that these changes led to an instrument that better captures safety climate/culture in the context of home care. In addition, the experts suggested adding the dimension of reporting client safety events from the SOPS HSOPSC 2.0 instrument (Agency for Healthcare Research and Quality 2019), as reporting events were considered an important aspect of safety culture. It was also considered to have a positive effect in increasing awareness for the reporting of client safety events, thus supporting a reporting culture in home care.

One further adaptation made was the addition of one open‐ended question asking what participants would recommend for improving client safety in their home care organisation. This item provides the opportunity for participants to make suggestions for improvements and might better inform strategies for changing safety culture. We suggest that it should be followed by discussion with the participants (e.g., interviews, focus groups), team reflection (e.g., using the Manchester patient safety framework (University of Manchester 2006)) and the involvement of the team in the design of strategies to improve safety culture. This adapted instrument includes items specific to the home care setting not existing in the original NHSOPSC, specifically on coordination within the organisation and with external healthcare/service providers and loss of information due to clients seeing different staff members. Based on expert input, we also added items on the provision of specific training on client safety and adequate resources; integration of lessons learned from errors; and provision of support for staff involved in safety errors to better reflect the reality of home care in Switzerland.

In comparison to two studies using the NHSOPSC in nursing homes (Cappelen et al. 2016; Vrotsou et al. 2021), our sample showed higher percentages of ‘Does not apply or don't know’ answers. However, our sample also consisted of more participants who had been working in the organisation for less than a year, were administration or technical staff, and compared with Cappelen et al. (2016), had also a higher percentage of participants who were not working directly with clients most of the time. In our sample, these characteristics, together with a percentage of employment ≤ 50%, were associated with a higher percentage of ‘Does not apply or don't know’ responses. Nevertheless, we believe that it is important to administer the survey to all home care staff, even if not all will be able to answer every item, as safety culture also reflects a group‐level concept based on shared values, beliefs and norms that influence everyone's safety behaviours. In addition, it is important to raise awareness for safety among all staff within the organisation.

Concerning reliability, in our study, the compliance with procedures dimension showed low internal consistency. This might be explained by the fact that the dimension had only two items, as we excluded one item (*Staff use shortcuts to get their work done faster*) from the original instrument. We didn't include this item as it was seen by the experts as a mechanism to deal with high workloads that does not necessarily always compromise client safety. In a previous study conducted in home care agencies, compliance with procedures also showed a Cronbach's alpha below 0.70 (Viksveen et al. 2022). Despite the low value (0.39), we decided to retain these items because in the expert consultations, it was considered an important aspect of safety culture. However, given the low internal consistency, it might be worthwhile to consider analysing the items as single items rather than as a dimension.

As many authors calculate the percentage of positive answers (or mean score) per dimension and an overall safety culture score (over all dimensions), we conducted single‐order and second‐order confirmatory factor analysis. The models showed acceptable fit. However, the evaluation of the discriminant validity and intercorrelations between the instrument's dimensions showed high correlations between three dimensions. A previous study reported that the CFA showed a negative definite matrix for some of the latent factors caused by almost perfect correlations between the same three dimensions. The authors merged these three dimensions into one dimension (Cappelen et al. 2016). In our case, the CFA model did not produce a negative definite matrix, indicating that the model estimation was stable, and the correlations, while high, did not reach problematic levels. For this reason, we decided to keep the three dimensions separate.

### 5.1 Strengths and Limitations

We used a stepwise approach involving patient safety culture and home care experts, as well as home care workers, which allowed a more comprehensive adaptation of the instrument to the Swiss home care setting. For the first time, a validated instrument exists that includes specific items to assess patient safety culture in the home care setting. Its adaptation resulted from close collaboration with experts and home care agencies and filled a gap in the assessment of patient safety culture in this context. Another strength is the addition of one additional open‐ended question to assess staff recommendations for improving client safety in their home care organisation.

The sample size was sufficient to ensure stable parameter estimates in the confirmatory factor analysis.

Because we only included two home care agencies, we didn't conduct multilevel confirmatory factor analysis. Our sample was a convenience sample, which may have introduced sampling bias and limit the generalisability of the results. In addition, although we followed the 10:1 respondent‐to‐item ratio rule of thumb, the resulting sample size may not have provided adequate statistical power, according to more recent evidence (Kline 2023). All variables were collected through self‐report survey, which may introduce common method bias and inflate observed correlations. Although the instrument includes open‐ended questions that allow respondents to provide more detailed information, it may not be sufficient to fully capture safety culture. Its assessment should be complemented by qualitative methods, such as focus groups, group reflections, interviews or observations. Together with the collection and analysis of other safety measures (e.g., safety events) and patient‐reported experiences, this could provide deeper insights into client safety.

### 5.2 Recommendations for Further Research

The adapted instrument has more items compared to the original NHSOPSC instrument. Although the instrument only takes about 15–20 min to complete, future research could evaluate the possibility of shortening the instrument without compromising the information it provides. Regarding its content, the three dimensions that showed high correlations should be investigated more closely to clarify whether the dimensions are truly distinct or should rather be shortened and merged into one dimension. Further psychometric testing should include a more thorough assessment of the convergent and discriminant validity of the instrument, as well as the reproducibility of the instrument (through test–retest reliability) in a longitudinal design. Furthermore, future studies should examine the instrument's responsiveness to change, that is, its ability to detect changes in safety climate following targeted interventions. To enable national‐level evaluation, the instrument should be translated, culturally adapted and validated for the other language regions of Switzerland.

### 5.3 Implications for Policy and Practice

The adaptation of the NHSOPSC to the home care setting provides managers with a valid and reliable tool to evaluate patient safety culture/climate in home care, enabling both its monitoring and the development of targeted interventions. In addition, the instrument can be used to raise awareness among home care workers and to initiate discussions about safety culture. To further cultivate safety culture, governments should incentivise its evaluation across all healthcare settings. National‐level use of the same instrument in home care would enable organisations to compare results and learn from each other.

## 6 Conclusion

Through a stepwise approach, we thoroughly adapted an instrument for the first time to the specific characteristics of the home care setting. The adapted instrument showed to be a valid and reliable instrument for measuring patient safety climate in Swiss French‐speaking home care agencies. This assessment—along with the interpretation of results and reflection with the participants, additional qualitative evaluations of safety culture and the collection of other safety measures and patient‐reported experiences—is essential for co‐designing strategies to improve safety culture. The translation, cultural adaptation and psychometric evaluation in home care in other countries and language regions would allow testing of the instrument's cross‐cultural validity.

## Author Contributions

Tania Martins: conceptualisation, methodology, formal analysis, writing – original draft, review and editing. Julie Bucher Andary: conceptualisation, writing – review and editing. David Bellagamba: conceptualisation, writing – review and editing. Michael Simon: conceptualisation, writing – review and editing. Franziska Zúñiga: conceptualisation, methodology, supervision, writing – review and editing. All authors read and approved the final manuscript.

## Funding

This work was supported by *Stiftung Pflegewissenschaft Schweiz* (Nursing Science Foundation Switzerland) (ID 3253‐2023). The funder was not involved in any phase of the research process.

## Disclosure

Statistical guidelines: The authors have checked to make sure that our submission conforms as applicable to the Journal's statistical guidelines. The statistics were checked prior to submission by an expert statistician (Kris Denhaerynck, kris.denhaerynck@unibas.ch). The authors affirm that the methods used in the data analyses are suitably applied to their data within their study design and context, and the statistical findings have been implemented and interpreted correctly. The authors agree to take responsibility for ensuring that the choice of statistical approach is appropriate and is conducted and interpreted correctly as a condition to submit to the Journal.

## Conflicts of Interest

The authors declare no conflicts of interest.

## Supporting information


**Appendix S1:** jan70479‐sup‐0001‐AppendixS1.docx.

**Appendix S2:** jan70479‐sup‐0002‐AppendixS2.docx.

**Appendix S3:** jan70479‐sup‐0003‐AppendixS3.docx.

**Appendix S4:** jan70479‐sup‐0004‐AppendixS4.docx.

**Appendix S5:** jan70479‐sup‐0005‐AppendixS5.docx.

**Appendix S6:** jan70479‐sup‐0006‐AppendixS6.docx.

**Data S1:** jan70479‐sup‐0007‐Supinfo.docx.

## Data Availability

Due to the sensitivity of the data analysed in this study, they are not publicly available, but are available from the corresponding author upon reasonable request.
