# Supplementary material for: Adaptation and Psychometric Evaluation of a Patient Safety Culture Instrument for Home Care—A Multicentre Cross‐Sectional Study
Source: J Adv Nurs. 2026 Jan 9;82(9):8603–20. doi: 10.1111/jan.70479 (PMC13460978; doi:10.1111/jan.70479)
Supplement: Supplementary file 2 [file JAN-82-8603-s002.docx]

**Appendix S2**

**Adaptations made to the NHSOPSC (Sorra et al., 2008) to measure safety culture in home care**

Overall changes:

- ‘*nursing home’* was replaced by ‘*organization*’ and ‘r*esidents*’ by ‘*clients*.’
- To avoid repeating “*in this organization*” in each question, we included it, where it was appropriate, at the beginning of the section. For example:

*‘In this organization …*

*T1. … staff treat each other with respect.’*

- At the beginning of the section, we added an introductory note explaining what “organization/center” and “supervisor” refer to.

Table S2 shows the adapted items and adaptations made following expert consultation, content validation, and cognitive interviews.

**Table S2.**

*Adaptations made to the NHSOPSC to measure safety culture/climate in home care*

| **Adapted items for home care** | **Adaptations made after expert consultation** | **Adaptations made after content validation** | **Adaptations made after cognitive interviews** |
| --- | --- | --- | --- |
| **Teamwork** | - In item T4, the following examples for ‘*helping out’* were provided in brackets ‘*(e.g., by taking over a visit, a service, or other duties).’* - One new item (T5) was added to cover the team coordination, as this was pointed out as an important aspect of teamwork in home care (adapted from SAQ-SF (Sexton et al., 2006)). |  |  |
| T1. In this organization, staff treat each other with respect. |  | - To what ‘*organization*’ refers to was added. | - – |
| T2. In this organization, staff support one another. |  |  |  |
| T3. In this organization, staff feel they are part of a team. |  |  |  |
| T4. In this organization, when someone gets really busy, the other team members are ready to help out (e.g., by taking over a visit, a service or other duties). |  |  |  |
| T5. In this organization, staff work together as a well-coordinated team. |  |  |  |
| **Staffing** | - NHSOPSC original item A16 (‘… *needs are met during* *shift changes’*) was adapted as shift changes were considered not relevant for the context of the Swiss home care as there are no formal shift changes. Instead, we assessed (item S3) overall if staff have enough time to respond to clients’ needs (adapted from the Safety Climate in Healthcare Organizations survey (Singer et al., 2007)),). | - In item S1, ‘*we’* was changed to ‘*staff’*. - In item S4 (NHSOPSC original item A17), ‘*staff* *quit their jobs’* was changed to ‘*of a high turnover rate’,* as turnover was considered a more practical manifestation of the problem of staff quitting their jobs. A definition of turnover was added as an information bullet. | - In item S2, the word ‘*generally’* was added to clarify that the statement refers to the general case. |
| S1. In this organization, there is enough staff to handle the workload. |  |  |  |
| S2. In this organization, staff generally have to hurry because they have too much work to do. (negatively worded) |  |  |  |
| S3. In this organization, staff have enough time to respond to clients' needs. |  |  |  |
| S4. In this organization, it's hard to keep clients safe because of a high turnover rate. (negatively worded) |  |  |  |
| **Compliance with procedures** | - In item C1, ‘*to care for clients’* was added to increase clarity. - NHSOPSC original item A6 (‘*use shortcuts’*) was removed as it was considered a repetition of items C1 and C2. - In item C2, the original item was rephrased to begin with ‘*staff often ignore procedures to get the work done easier’.* | - Item C1, examples of procedures were added to clarify what is meant by ‘*procedures*’. - In item C2, the item text was reverted to its original version, beginning with ‘*to get the work done easier’* as it was considered clearer. |  |
| C1. In this organization, staff follow the procedures in place (e.g., procedure in the event of a fall, death, etc.) to care for clients. |  |  |  |
| C2. In this organization, to get the work done easier, staff often ignore the procedures in place in the organization. (negatively worded) |  |  |  |
| **Training and skills** | - In the original NHSOPSC item A11 (‘… *have enough training on how to handle difficult residents’),* ‘*difficult residents’* was changed to ‘*difficult client situations’* (Z2), as the original phrasing might suggest that clients themselves are difficult, rather than the situations. - The original NHSOPSC item A13 (‘*understand the training they get’*) was excluded, since it was considered likely to be interpreted as an assessment of individuals’ capability to understand the training provided. In its place, we added a new item (Z3) on ‘*getting specific training in client safety’*. (Example of supporting literature: (Institute of Medicine (US) Committee on the Work Environment for Nurses and Patient Safety, 2004)). |  |  |
| Z1. In this organization, staff get the training they need to feel confident in the performance of their job. |  | - We revised item Z1 by adding ‘*to feel confident in the performance of their job’* to improve clarity on what the training refers to. - In item Z2, ‘*difficult client situations’*, was reworded to ‘*difficult situations with clients’* to enhance clarity. | - A information bullet point was added to ‘*client safety’*, as the meaning of the concept was not clear to all respondents. |
| Z2. In this organization, staff have enough training on how to handle difficult situations with the clients. |  |  |  |
| Z3. In this organization, staff get specific training in client safety. |  |  |  |
| **Nonpunitive response to mistakes** | - A new item ‘*When staff makes errors, this organization focuses on learning rather than blaming individuals’* (item R1) (adapted from HSOPSC version 2.0 (Agency for Healthcare Research and Quality, 2019)) was added. - A new item (R5), ‘*staff involved in client safety errors do not receive sufficient support,’* was added to address the topic of second victim support. (Examples of supporting literature: (Agency for Healthcare Research and Quality, 2019; John et al., 2024)). - In item R6, a definition of ‘*harm*’ was added. | - *–* | - *–* |
| R1. In this organization, when staff makes errors, this organization focuses on learning rather than blaming individuals. |  |  |  |
| R2. In this organization, staff are afraid to report their mistakes. (negatively worded) |  |  |  |
| R3. In this organization, staff are treated fairly when they make mistakes. |  |  |  |
| R4. In this organization, staff feel safe reporting their mistakes. |  |  |  |
| R5. In this organization, staff involved in client safety errors do not receive sufficient support. (negatively worded) |  |  |  |
| R6. In this organization, staff are blamed when a client is harmed. (negatively worded) |  |  |  |
| **Handoffs and information exchange** | “Information Exchange” was added to the domain title, as it was considered more appropriate for the home care setting and the domain’s items. |  |  |
| H1. Staff have all the information they need to know before taking care of a new client for the first time. | - In item H1, ‘*are told’* was replaced by ‘*have all the information’,* to better reflect the reality of Swiss home care, where information exchange occurs mainly through the electronic health record. - In item H2, ‘*are told’* was changed to *‘are notified’* to better reflect the reality of Swiss home care, as information exchange mainly occurs in written form. - Item H3 was adapted to include healthcare facilities other than hospitals. - In item H4, ‘*are given’* was changed to *‘have’ to* better reflect the reality of Swiss home care. - A new item (H6) was added to assess handoffs between teams within the home care organization (Example of supporting literature: SCOPE-PC (Desmedt et al., 2018)). - A new item (H7) was added to assess information loss when clients are care for by different staff members (adapted from the SCOPE-PC (Desmedt et al., 2018)). | - In item H1, *new* was added to make clear that it’s about a new client. - In item H2, ‘*before the visit’* was added in place of ‘*tight away’,* as this was considered the moment when the information needs to be available. | - Item H3 was divided in two items to separately assess handoffs after inpatient care (hospitals, nursing homes, etc.) and outpatient care (medical consultations (GP, specialists), resulting in items H3 and H5. |
| H2. Staff are notified when there is a change in a client’s care plan/service provision before the visit. |  |  |  |
| H3. Staff have all the information they need when clients are transferred from other healthcare facilities (hospitals, nursing homes, etc.). |  |  |  |
| H4. Staff have all the information they need to care for clients. |  |  |  |
| H5. Staff have all the information they need after clients had a medical consultation (general practitioner, specialist, etc.). |  |  |  |
| H6. Staff have all the information they need when clients are transferred from one team to another within the organization. |  |  |  |
| H7. In this organization, important information is often lost because clients see different staff. |  |  |  |
| **Feedback and communication about incidents** |  |  |  |
| F1. In this organization, when staff report something that could harm a client to their manager, someone in the organization takes care of it. | - In item F3, *‘someone’* was replaced with ‘*their manager’*, as ‘*someone’* was considered too vague. - In item F2, ‘*talk’* was changed to ‘*reflect,’* as in French this was considered the more culturally appropriate term. - In item F4, ‘*keep residents safe from* harm’ was culturally adapted to ‘*ensure clients' safety,’* as this wording was considered clearer. - A new Item (F5) was added to assess whether changes made based on incident reports are communicated to staff (adapted from HSOPSC 2.0 (Agency for Healthcare Research and Quality, 2019)). | - In item F1, ‘*to their manager’* and ‘*of the organization’* were added to increase clarity. - In items F4 and F5, ‘*we’* was changed to ‘*staff’ for consistency.* - In item F5, the French word for *reports* was changed (from ‘*déclarations’* to ‘*signalement’*). | - – |
| F2. In this organization, we reflect together about ways to keep incidents from happening again. |  |  |  |
| F3. In this organization, staff tell their manager if they see something that might harm a client. |  |  |  |
| F4. In this organization, staff discuss ways to ensure clients' safety. |  |  |  |
| F5. In this organization, staff are informed about corrective measures or improvements made based on incident reports. |  |  |  |
| **Communication openness** |  |  |  |
| O1. In this organization, staff ideas and suggestions are valued. | - The original NHSOPSC item B9 (‘*staff opinions are ignored’*) was excluded, as experts considered it redundant with item B7 (adapted as item O1). - A new item (O3) was added to assess whether communication is founded on trust. - A new item O4 was added (‘*staff are afraid to ask questions when something does not seem right’*) (adapted from HSOPS 2.0 (Agency for Healthcare Research and Quality, 2019)), as it was considered relevant for evaluating communication openness. | - In item O1, the French word for ‘*valued*’ was changed from ‘*valorisées’* to ‘*considérées’*. | - – |
| O2 In this organization, it is easy for staff to speak up about problems. |  |  |  |
| O3. In this organization, communication is founded on trust. |  |  |  |
| O4. In this organization, staff are afraid to ask questions when something does not seem right. (negatively worded) |  |  |  |
| **Supervisor or manager expectations and actions promoting client safety** | - In item P3, ‘*pays attention’* was changed to ‘*takes action,’* as the domain assesses expectations and actions. - A new item (P4) was added (‘*my supervisor is actively committed to client safety and thus serves as a role model’*) to explicitly assess whether supervisors demonstrate commitment to client safety and act as role models (Example of supporting literature (Bisbey et al., 2021)). | - In item P4, ‘*and thus serves as a role model’* was deleted, as it was considered redundant; if a supervisor or manager is committed, they are already acting as a role model. |  |
| P1. My supervisor or manager listens to staff ideas and suggestions about client safety. |  |  | - – |
| P2. My supervisor or manager expresses satisfaction when work is carried out in compliance with safety procedures. |  |  |  |
| P3. My supervisor or manager takes action to address client safety concerns that are brought to their attention. |  |  |  |
| P4. My supervisor or manager is actively committed to client safety. |  |  |  |
| **Overall perceptions of client safety** |  |  |  |
| B1. Clients are well cared for in this organization. | - The original NHSOPSC item D8 (‘*this nursing home is a safe place for residents’*) was changed to ‘*this organization provides safe care/services to clients’* (B3), as this wording was considered more appropriate for the home care setting. - A new item (B4) (‘*The safety of care/services is not neglected in favor of other interests that might affect client safety*) was added (adapted from the Swiss version of the Safety Climate Survey (Gehring et al., 2015)). - A new item (B5) was added to assess whether staff perceive that the organization is doing more for client safety now than one year ago (adapted from the Swiss version of the Safety Climate Survey (Gehring et al., 2015)) | - In item B4, ‘*other interest that might affect client safety’* was changed to ‘*greater productivity,’* as it was suggested to be easier to understand. | - – |
| B2. This organization does a good job to ensure clients safety. |  |  |  |
| B3. This organization provides safe care/services to clients. |  |  |  |
| B4. The safety of care/services is not neglected in favour of greater productivity. |  |  |  |
| B5. This organization is doing more for client safety now than it did one year ago. |  |  |  |
| **Management support for client safety** |  |  |  |
| M1. The direction listens to staff ideas and suggestions to improve client safety. | - Original NHSOPSC item D9 (‘*management often walks around the nursing home to check on resident care’*) was excluded, as it was not considered relevant for the home care setting. - Item M2 (‘*the actions of the management show that client safety is one of the top priorities’*) was newly added based on the expert’s feedback. - Item M3 (*direction seems interested in client safety only after an advent event happens*) was added. - Item M4 (‘*the direction provides adequate resources to improve client safety’*) was added (adapted from the Safety Climate in Healthcare Organizations survey (Singer et al., 2007)), as the availability of resources was considered an important aspect in providing care in clients’ homes. | - In item M3, the French word for ‘*adverse event’* was revised, replacing ‘*événement’* with *‘incident’*. - In item M4, examples of resources were added ‘*(e.g., materials, technological resources, human resources, etc.)’.* | - – |
| M2. The actions of the management show that client safety is one of the top priorities. |  |  |  |
| M3. The direction seems interested in client safety only after an adverse event happens. |  |  |  |
| M4. The direction provides adequate resources (e.g., materials, technological resources, human resources, etc.) to improve client safety. |  |  |  |
| M5. The direction asks staff how the organization can improve client safety. |  |  |  |
| **Organizational learning** |  |  |  |
| L1. In this organization, the same mistakes happen again and again. (negatively worded) | - No additional changes were made. | - – | - – |
| L2. In this organization, it is easy to make changes to improve client safety. |  |  |  |
| L3. This organization is always doing things to improve client safety. |  |  |  |
| L4. When this organization makes changes to improve client safety, it checks to see if the changes worked. |  |  |  |
| **Reporting client safety events** |  |  |  |
| A1. When a mistake is caught and corrected before reaching the client, it is reported.. | - This domain (originally from HSOPSC 2.0 (Agency for Healthcare Research and Quality, 2019)) was added to assess reporting of client safety events. | - In item A2, the French wording was revised, replacing ‘*erreur’* (mistake) with ‘*préjudice’* to better capture the intended meaning. | - The question mark and the wording “how often” were removed, and an “…” was added, as this wording was considered easier, shorter, and easier to understand. |
| A2. When a mistake reaches the client, but did not harm him, it is reported …. |  |  |  |
| **Overall ratings** |  |  |  |
| G2. I would tell friends that this is a safe home care organization for their family. | - In item G1, the sentence structure was revised, changing ‘*Please give this nursing home an overall rating* …’ to ‘*Overall, how would you rate .*..’ | - – | - – |
| G1. Overall, how do you rate the client safety in your organization? |  |  |  |
| **Open-ended questions** |  |  |  |
| D1. What are your recommendations for improving client safety in this home care organization? | - Item D2 was added, as it was considered important to assess staff views on measures for improvement (adapted from SAQ-SF (Sexton et al., 2006)). | - In item D2, the reference to ‘*three recommendations’* was removed, as it was regarded as potentially imposing a minimum or maximum number of recommendations. | - – |
| D2. Please feel free to write any comments about client care and/or safety in this home care organization. |  |  |  |
| **Background information** |  |  |  |
| I1. What is your main job/position in this home care organization? | - The NHSOPSC items ‘*Are you paid by a staffing agency when you work for this nursing home’* and ‘*In this nursing home, where do you spend most of the time working? (specific units)’* were not considered relevant for the Swiss home care setting and were therefore removed. |  |  |
| I2.In which year did you start working in this organization? |  |  |  |
| l3. What is your employment percentage of your main job in this organization? |  |  |  |
| I4. When do you work most often? Please choose only ONE option. |  |  |  |
| I5. In your staff position, do you typically have direct interaction or contact with clients? |  |  |  |

*Note.* NHSOPSC, Nursing Home Survey on Patient Safety Culture; HSOPSC, Hospital Survey on Patient Safety Culture; SAQ-SF, Safety Attitudes Questionnaire– Short Form; SCOPE-PC, SCOPE-Primary Care. This is not a validated English translation.

**References**

Agency for Healthcare Research and Quality. (2019). *Hospital Survey on Patient Safety Culture*. <https://www.ahrq.gov/sops/surveys/hospital/index.html>

Bisbey, T. M., Kilcullen, M. P., Thomas, E. J., Ottosen, M. J., Tsao, K., & Salas, E. (2021). Safety Culture: An Integration of Existing Models and a Framework for Understanding Its Development. *Hum Factors*, *63*(1), 88-110. <https://doi.org/10.1177/0018720819868878>

Desmedt, M., Bergs, J., Willaert, B., Vlayen, A., Hellings, J., Schrooten, W., . . . Vandijck, D. (2018). The SCOPE-PC instrument for assessing patient safety culture in primary care: a psychometric evaluation. *Acta Clin Belg*, *73*(2), 91-99. <https://doi.org/10.1080/17843286.2017.1344760>

Gehring, K., Mascherek, A. C., Bezzola, P., & Schwappach, D. L. (2015). Safety climate in Swiss hospital units: Swiss version of the Safety Climate Survey. *J Eval Clin Pract*, *21*(2), 332-338. <https://doi.org/10.1111/jep.12326>

Institute of Medicine (US) Committee on the Work Environment for Nurses and Patient Safety. (2004). *Keeping Patients Safe: Transforming the Work Environment of Nurses.* <https://www.ncbi.nlm.nih.gov/books/NBK216181/>

John, M., Joann, S., Bryan, G., & Sarah, M. (2024). Ensuring Patient and Workforce Safety Culture in Healthcare. *PSNet [internet]*.

Sexton, J. B., Helmreich, R. L., Neilands, T. B., Rowan, K., Vella, K., Boyden, J., . . . Thomas, E. J. (2006). The Safety Attitudes Questionnaire: psychometric properties, benchmarking data, and emerging research. *BMC Health Services Research*, *6*(1), 44. <https://doi.org/10.1186/1472-6963-6-44>

Singer, S., Meterko, M., Baker, L., Gaba, D., Falwell, A., & Rosen, A. (2007). Workforce perceptions of hospital safety culture: development and validation of the patient safety climate in healthcare organizations survey. *Health Serv Res*, *42*(5), 1999-2021. <https://doi.org/10.1111/j.1475-6773.2007.00706.x>

Sorra, J., Franklin, M., & Streagle, S. (2008). Nursing home survey on patient safety culture. *Rockville: Agency for Healthcare Research and Quality*, 08-0060. <https://www.ahrq.gov/sites/default/files/publications/files/nhguide.pdf>
