# Supplementary material for: Adaptation and Psychometric Evaluation of a Patient Safety Culture Instrument for Home Care—A Multicentre Cross‐Sectional Study
Source: J Adv Nurs. 2026 Jan 9;82(9):8603–20. doi: 10.1111/jan.70479 (PMC13460978; doi:10.1111/jan.70479)
Supplement: Supplementary file 3 [file JAN-82-8603-s003.docx]

**Appendix S5**

**One-factor CFA models**

**Table S5.**

*Results of one-factor CFA: Factor loadings and model fit.*

| **Dimension** | **Item** | **Factor loadings (standardized)** | **Model goodness-of-fit** |
| --- | --- | --- | --- |
| Teamwork | T1 | 0.847 | χ2 (df =5, n = 672) = 14.33, *p* = 0.014  CFI = 0.992  TLI = 0.985  RMSEA = 0.08 (90% CI: 0.06, 0.11)  SRMR = 0.032 |
|  | T2 | 0.825 |  |
|  | T3 | 0.773 |  |
|  | T4 | 0.550 |  |
|  | T5 | 0.846 |  |
| Staffing | S1 | 0.715 | χ2 (df =2, n = 671) = 6.10, *p* = 0.047  CFI: 0.993  TLI: 0.978  RMSEA: 0.09 (90% CI 0.05, 0.14)  SRMR: 0.024 |
|  | S2 | 0.693 |  |
|  | S3 | 0.838 |  |
|  | S4 | 0.640 |  |
| Compliance with procedures | C1 | 0.879 | Does not apply |
|  | C2 | 0.400 |  |
| Training and skills | Z1 | 0.636 | Does not apply |
|  | Z2 | 0.604 |  |
|  | Z3 | 0.892 |  |
| Nonpunitive response to mistakes | R1 | 0.597 | χ2 (df =5, n = 656) = 36.14, *p* = 0.000  CFI:0.966  TLI: 0.931  RMSEA:0.14 (90% CI: 0.11, 0.17)  SRMR:0.049 |
|  | R2 | 0.623 |  |
|  | R3 | 0.758 |  |
|  | R4 | 0.821 |  |
|  | R5 | 0.622 |  |
| Handoffs and information exchange | H1 | 0.837 | χ2 (df =9, n = 651) = 19.71, *p* = 0.020  CFI: 0.992  TLI: 0.987  RMSEA: 0.07 (90% CI 0.05, 0.10)  SRMR: 0.029 |
|  | H2 | 0.621 |  |
|  | H3 | 0.825 |  |
|  | H4 | 0.840 |  |
|  | H5 | 0.788 |  |
|  | H6 | 0.720 |  |
| Feedback and communication | F1 | 0.667 | χ2 (df =5, n = 654) = 25.38, *p* = 0.000  CFI:0.984  TLI:0.969  RMSEA:0.11 (90% CI 0.09, 0.14)  SRMR:0.039 |
|  | F2 | 0.776 |  |
|  | F3 | 0.580 |  |
|  | F4 | 0.752 |  |
|  | F5 | 0.699 |  |
| Communication openness | O1 | 0.739 | Does not apply |
|  | O2 | 0.712 |  |
|  | O3 | 0.904 |  |
| Supervisor or manager expectations and actions promoting client safety | P1 | 0.889 | χ2 (df =2, n = 655) = 14.37, *p*= 0.001  CFI:0.997  TLI:0.992  RMSEA: 0.14 (90% CI: 0.09, 0.18)  SRMR: 0.026 |
|  | P2 | 0.825 |  |
|  | P3 | 0.952 |  |
|  | P4 | 0.935 |  |
| Overall perceptions of client safety | B1 | 0.861 | χ2 (df =2, n = 660) = 0.177, *p*= 0.915  CFI:1.00  TLI: 1.00  RMSEA: 0.00 (90% CI 0.00, 0.53)  SRMR:0.004 |
|  | B2 | 0.891 |  |
|  | B3 | 0.891 |  |
|  | B4 | 0.621 |  |
| Management support for client safety | M1 | 0.834 | χ2 (df =5, n = 659) = 19.01, *p*= 0.002  CFI: 0.992  TLI: 0.984  RMSEA: 0.11 (90% CI 0.08, 0.14)  SRMR: 0.028 |
|  | M2 | 0.855 |  |
|  | M3 | 0.626 |  |
|  | M4 | 0.802 |  |
|  | M5 | 0.830 |  |
| Organizational learning | L1 | 0.699 | χ2 (df =2, n = 653) = 19.01, *p*= 0.215  CFI: 0.995  TLI: 0.986  RMSEA:0.07 (90% CI 0.02, 0.12)  SRMR:0.014 |
|  | L2 | 0.809 |  |
|  | L3 | 0.913 |  |
|  | L4 | 0.803 |  |
| Reporting client safety events | A1 | 0.782 | Does not apply |
|  | A2 | 0.884 |  |

*Note*. X2, Chi-square; df, degrees of freedom; *p*, *p*-value; CFI, comparative fit index, TLI, Tucker-Lewis Index; RMSEA, root mean square error of approximation; SRMR, standardized root mean square residual; CI, confidence interval.
