# Supplementary material for: Adaptation and Psychometric Evaluation of a Patient Safety Culture Instrument for Home Care—A Multicentre Cross‐Sectional Study
Source: J Adv Nurs. 2026 Jan 9;82(9):8603–20. doi: 10.1111/jan.70479 (PMC13460978; doi:10.1111/jan.70479)
Supplement: Supplementary file 4 [file JAN-82-8603-s004.docx]

**Appendix S3**

**Patient Safety Culture Survey for Home Care (PSCS–HC)**

| **Section A: Working in this home care organization**  *For the purpose of this survey, the term ‘organization’ refers to the hierarchical structure that most directly concerns you. The term ‘manager’ refers to the person who directly supervises your work (e.g., team leader, manager, director).*  How much do you agree or disagree with the following statements about your home care organization? | | |
| --- | --- | --- |
| In this organization … | | |
| T1. | ... staff treat each other with respect. | 1- Strongly disagree 2- Disagree 3- Neither agree nor disagree 4- Agree 5- Strongly agree   10- Does not apply or don’t know |
| T5. | ... staff work together as a well-coordinated team. |  |
| S1. | ... there is enough staff to handle the workload. |  |
| T2. | ... staff support one another. |  |
| In this organization, staff ... | | |
| C1. | ... follow the procedures in place (e.g., procedure in the event of a fall, death, etc.) to care for clients. | 1- Strongly disagree 2- Disagree 3- Neither agree nor disagree 4- Agree 5- Strongly agree   10- Does not apply or don’t know |
| T3. | ... feel they are part of a team. |  |
| Z1. | ... get the training they need to feel confident in the performance of their job. |  |
| S2. | ... generally, have to hurry because they have too much work to do. |  |
| In this organization ... | | |
| T4. | ... when someone gets really busy, the other team members are ready to help out (e.g., by taking over a visit, a service or other duties). | 1- Strongly disagree 2- Disagree 3- Neither agree nor disagree 4- Agree 5- Strongly agree   10- Does not apply or don’t know |
| R6. | ... staff are blamed when a client is harmed. |  |
| Z2. | ... staff have enough training on how to handle difficult situations with the clients. |  |
| R2. | ... staff are afraid to report their mistakes. |  |
| R1. | ... when staff makes errors, this organization focuses on learning rather than blaming individuals. |  |
| In this organization ... | | |
| Z3. | ... staff get specific training in client safety. | 1- Strongly disagree 2- Disagree 3- Neither agree nor disagree 4- Agree 5- Strongly agree   10- Does not apply or don’t know |
| C2. | ... to get the work done easier, staff often ignore the procedures in place in the organization. |  |
| R3. | ... staff are treated fairly when they make mistakes. |  |
| S3. | ... staff have enough time to respond to clients' needs. |  |
| S4. | ... it's hard to keep clients safe because of a high turnover rate. |  |
| R4. | ... staff feel safe reporting their mistakes. |  |
| R5. | ... staff involved in client safety errors do not receive sufficient support. |  |
| **Section B: Communication**  *For the purpose of this survey, the term ‘organization’ refers to the hierarchical structure that most directly concerns you* | | |
| How often do the following things happen in your home care organization? | | |
| H1. | Staff have all the information they need to know before taking care of a client for the first time. | 1- Never 2- Rarely 3- Sometimes 4- Most of the time 5- Always   10- Does not apply or don’t know |
| H2. | Staff are notified when there is a change in a client’s care plan/service provision before the visit. |  |
| H3. | Staff have all the information they need when clients are transferred from other healthcare facilities (hospitals, nursing homes, etc.). |  |
| F1. | In this organization, when staff report something that could harm a client to their manager, someone in the organization takes care of it. |  |
| F2. | In this homecare organization, we reflect together about ways to keep incidents from happening again. |  |
| H5. | Staff have all the information they need after clients had a medical consultation (general practitioner, specialist, etc.). |  |
| In this organization ... | | |
| F3. | ... staff tell their manager if they see something that might harm a client. | 1- Never 2- Rarely 3- Sometimes 4- Most of the time 5- Always   10- Does not apply or don’t know |
| O1. | ... staff ideas and suggestions are valued. |  |
| F4. | ... staff discuss ways to ensure clients’ safety. |  |
| F5. | ... staff are informed about corrective measures or improvements made based on incident reports. |  |
| O4. | … staff are afraid to ask questions when something does not seem right. |  |
| In this organization ... | | |
| H4. | ... staff have all the information they need to care for clients. | 1- Never 2- Rarely 3- Sometimes 4- Most of the time 5- Always   10- Does not apply or don’t know |
| H7. | … important information is often lost because clients see different staff. |  |
| O2. | ... it is easy for staff to speak up about problems. |  |
| O3. | ... communication is founded on trust. |  |
| H6. | ... staff have all the information they need when clients are transferred from one team to another within the organization. |  |
| **Section C: Your supervisor**  *For the purpose of this study the term ‘supervisor or manager’ refers to the person who directly supervises your work (e.g., team leader, manager, director).* | | |
| How much do you agree or disagree with the following statements? | | |
| My supervisor or manager ... | | |
| P1. | ... listens to staff ideas and suggestions about client safety. | 1- Strongly disagree 2- Disagree 3- Neither agree nor disagree 4- Agree 5- Strongly agree 10- Does not apply or don’t know |
| P2. | ... expresses satisfaction when work is carried out in compliance with safety procedures. |  |
| P3. | ... takes action to address client safety concerns that are brought to their attention. |  |
| P4. | ... is actively committed to client safety. |  |
| **Section D: Your home care organization** | | |
| How much do you agree or disagree with the following statements? | | |
| B1. | Clients are well cared for in this organization. | 1- Strongly disagree 2- Disagree 3- Neither agree nor disagree 4- Agree 5- Strongly agree   10- Does not apply or don’t know |
| M1. | The direction listens to staff ideas and suggestions to improve client safety. |  |
| M5. | The direction asks staff how the organization can improve client safety. |  |
| L1. | In this organization, the same mistakes happen again and again. |  |
| L2. | In this organization, it is easy to make changes to improve client safety. |  |
| L3. | This organization is always doing things to improve client safety. |  |
| B2. | This organization does a good job to ensure clients safety. |  |
| M2. | The actions of the management show that client safety is one of the top priorities. |  |
| B3. | This organization provides safe care/services to clients. |  |
| M3. | The direction seems interested in client safety only after an adverse event happens. |  |
| M4. | The direction provides adequate resources (e.g., materials, technological resources, human resources, etc.) to improve client safety. |  |
| L4. | When this organization makes changes to improve client safety, it checks to see if the changes worked. |  |
| B4. | The safety of care/services is not neglected in favor of greater productivity. |  |
| B5. | This organization is doing more for client safety now than it did one year ago. |  |
| **Section E: Reporting client safety events** | | |
| How often do the following things happen in your home care organization? | | |
| A1. | When a mistake is caught and corrected before reaching the client, it is reported.... | 1- Never 2- Rarely 3- Sometimes 4- Most of the time 5- Always   10- Does not apply or don’t know |
| A2. | When a mistake reaches the client, but did not harm him, it is reported... |  |
| A3. | In the past 12 months, how many client safety events have you reported? | 1- None 2- 1 to 2 3- 3 to 5 4- 6 to 10 5- 11 or more |
| In this organization... | | |
| A5. | ... does an anonymous IT tool exist for reporting client safety problems? | 1- Yes 2- Partially/incompletely present 3- No   10- Don’t know |
| A6. | … are there clear procedures for reporting and dealing with client safety incidents? |  |
| **Section F: Overall ratings**  *“Client safety” is defined as the avoidance and prevention of client harm/injuries or adverse events resulting from healthcare services provided to clients.* | | |
| G1. | Overall, how do you rate the client safety in your organization? | 1- Poor 2- Fair 3- Good 4- Very good 5- Excellent |
| G2. | I would tell friends that this is a safe home care organization for their family. | 1- Yes 2- Maybe 3- No |
| D1. | What are your recommendations for improving client safety in your home care organization? | |
|  |  | |
| D2. | Please feel free to write any comments about client care and/or safety in your home care organization. | |
|  |  | |
| **Background questions** | | |
| 1. | What is your main job/position in this home care organization? | 1- Registered Nurse (RN) 2- Licensed Practical Nurse (LPN), Licensed Vocational Nurse (LVN) 3- Administrative support staff, technical staff 4- Clinical experts, clinical nurse specialist, nurse practitioner, consultant 5- Care coordinator, planner 6- Social worker, dietitian, occupational therapist, physiotherapist, pharmacist 7- Director, administrator,   supervisor, manager   8- Nursing assistant, nursing aide*, s*ocio-educational support, housekeeping, health and social care assistant 9- Student, apprentice, trainee 10- Other (Please write the title of your job): _______________ |
| 2. | In which year did you start working in this organization? | ––––––––– |
| 3. | What is your employment percentage of your main job in this organization? | %: __________ |
| 4. | When do you work most often? Please choose only ONE option. | 1- Mornings 2- Evenings 3- Nights 4- Mornings and evenings 5- Mornings and nights 6- Evenings and nights 7- Mornings, evenings and nights |
| 5. | In your staff position, do you typically have direct interaction or contact with clients? | 1- Yes  2- No |

*Note.* This is an unofficial translation of the French version of the instrument.

© 2025. This work is licensed under [CC BY-NC-SA 4.0.](https://creativecommons.org/licenses/by-nc-sa/4.0/?ref=chooser-v1)
