# Supplementary material for: Adaptation and Psychometric Evaluation of a Patient Safety Culture Instrument for Home Care—A Multicentre Cross‐Sectional Study
Source: J Adv Nurs. 2026 Jan 9;82(9):8603–20. doi: 10.1111/jan.70479 (PMC13460978; doi:10.1111/jan.70479)
Supplement: Supplementary file 5 [file JAN-82-8603-s005.docx]

**Appendix S1**

**Table S1.**

*Content Validity Index of the adapted version of the NHSOPSC for home care. The content validity was evaluated by 9 experts.*

| **Dimensions / Items** | **I-CVI** | **S-CVI/ Ave** |
| --- | --- | --- |
| **Teamwork** |  | 0.67 |
| Item T1 | 0.78 |  |
| Item T2 | 0.67* |  |
| Item T3 | 0.56* |  |
| Item T4 | 0.78 |  |
| Item T5 | 0.55* |  |
| **Staffing** |  | 0.80 |
| Item S1 | 1.00 |  |
| Item S2 | 0.78 |  |
| Item S3 | 0.89 |  |
| Item S4 | 0.55 |  |
| **Compliance with procedures** |  | 0.82 |
| Item C1 | 0.89 |  |
| Item C2 | 0.89 |  |
| Item C3^a^ | 0.67 |  |
| **Training and skills** |  | 0.82 |
| Item Z1 | 0.78 |  |
| Item Z2 | 0.89 |  |
| Item Z3 | 0.78 |  |
| **Nonpunitive response to mistakes** |  | 0.93 |
| Item R1 | 1.00 |  |
| Item R2 | 0.89 |  |
| Item R3 | 0.89 |  |
| Item R4 | 1.00 |  |
| Item R5 | 0.89 |  |
| Item R6^b^ | 0.89 |  |
| **Handoffs and information exchange** |  | 0.89 |
| Item H1 | 0.88 |  |
| Item H2 | 0.78 |  |
| Item H3 | 1.00 |  |
| Item H4 | 1.00 |  |
| Item H6 | 0.89 |  |
| Item H7^b^ | 0.78 |  |
| **Feedback and communication about incidents** |  | 0.96 |
| Item F1 | 0.78 |  |
| Item F2 | 1.00 |  |
| Item F3 | 1.00 |  |
| Item F4 | 1.00 |  |
| Item F5 | 1.00 |  |
| **Communication openness** |  | 0.95 |
| Item O1 | 1.00 |  |
| Item O2 | 1.00 |  |
| Item O3 | 1.00 |  |
| Item O4^b^ | 0.78 |  |
| **Supervisor or manager expectations and actions promoting client safety** |  | 0.97 |
| Item P1 | 1.00 |  |
| Item P2 | 1.00 |  |
| Item P3 | 1.00 |  |
| Item P4 | 0.89 |  |
| **Overall perceptions of client safety** |  | 0.82 |
| Item B1 | 0.89 |  |
| Item B2 | 0.67^#^ |  |
| Item B3 | 1.00 |  |
| Item B4 | 0.67 |  |
| Item B5^b^ | 0.89 |  |
| **Management support for client safety** |  | 0.87 |
| Item M1 | 1.00 |  |
| Item M2 | 0.89 |  |
| Item M3 | 0.78 |  |
| Item M4 | 0.78 |  |
| Item M5 | 0.89 |  |
| **Organizational learning** |  | 0.95 |
| Item L1 | 0.89 |  |
| Item L2 | 0.89 |  |
| Item L3 | 1.00 |  |
| Item L4 | 1.00 |  |
| **Reporting client safety events** |  | 0.95 |
| Item A1 | 1.00 |  |
| Item A2 | 0.89 |  |

*Note.* Nine experts rated the relevance of each item on a 4-point scale from 1=’not relevant’ to 4=’highly relevant’. The item-level content validity index (I-CVI) was calculated as the proportion of experts rating an item as either 3 or 4. Items with I-CVI <0.78 (Polit et al., 2007) were reviewed or removed. All domains were judged to be both relevant and comprehensive.

^a^This item was removed after the content validation.

^b^These items (R6, H7, O4, B5) were later removed from the instrument.

*Although the I-CVI was < 0.78, this value resulted from a misunderstanding between the terms *team*, *organization,* and *center*. We addressed this by adding an introductory note that defines each concept.

^#^Although the I-CVI was < 0.78, this was due to the perception that the term ‘*good*’ is too subjective. Nevertheless, we decided to retain the word, as the aim is to allow individual interpretations of ‘*good*’.
