# Supplementary material for: Adaptation and Psychometric Evaluation of a Patient Safety Culture Instrument for Home Care—A Multicentre Cross‐Sectional Study
Source: J Adv Nurs. 2026 Jan 9;82(9):8603–20. doi: 10.1111/jan.70479 (PMC13460978; doi:10.1111/jan.70479)
Supplement: Supplementary file 6 [file JAN-82-8603-s006.docx]

**Appendix S6**

**Intercorrelations between instruments’ dimensions**

**Table S6.**

*Intercorrelations (Spearman’s rho) among the safety culture dimensions of the adapted instrument.*

|  | **Safety culture dimensions** | 1 | 2 | 3 | 4 | 5 | 6 | 7 | 8 | 9 | 10 | 11 | 12 |
| --- | --- | --- | --- | --- | --- | --- | --- | --- | --- | --- | --- | --- | --- |
| 1 | Teamwork | – |  |  |  |  |  |  |  |  |  |  |  |
| 2 | Staffing | 0.41 | – |  |  |  |  |  |  |  |  |  |  |
| 3 | Compliance with procedures | 0.33 | 0.32 | – |  |  |  |  |  |  |  |  |  |
| 4 | Training and skills | 0.45 | 0.38 | 0.31 | – |  |  |  |  |  |  |  |  |
| 5 | Nonpunitive response to mistakes | 0.51 | 0.44 | 0.37 | 0.42 | – |  |  |  |  |  |  |  |
| 6 | Handoffs and information exchange | 0.32 | 0.44 | 0.30 | 0.52 | 0.38 | – |  |  |  |  |  |  |
| 7 | Feedback and communication about incidents | 0.43 | 0.39 | 0.35 | 0.50 | 0.51 | 0.60 | – |  |  |  |  |  |
| 8 | Communication openness | 0.47 | 0.45 | 0.30 | 0.38 | 0.54 | 0.47 | 0.67 | – |  |  |  |  |
| 9 | Supervisor or manager expectations and actions promoting client safety | 0.42 | 0.32 | 0.27 | 0.41 | 0.46 | 0.42 | 0.55 | 0.55 | – |  |  |  |
| 10 | Overall perceptions of client safety | 0.48 | 0.53 | 0.36 | 0.53 | 0.50 | 0.53 | 0.61 | 0.58 | 0.59 | – |  |  |
| 11 | Management support for client safety | 0.42 | 0.51 | 0.37 | 0.53 | 0.49 | 0.57 | 0.61 | 0.54 | 0.59 | 0.73 | – |  |
| 12 | Organizational learning | 0.45 | 0.52 | 0.38 | 0.54 | 0.52 | 0.56 | 0.64 | 0.57 | 0.55 | 0.75 | 0.79 | – |
| 13 | Reporting client safety events | 0.28 | 0.26 | 0.31 | 0.40 | 0.37 | 0.51 | 0.58 | 0.43 | 0.36 | 0.42 | 0.43 | 0.46 |
