# Supplementary material for: Adaptation and Psychometric Evaluation of a Patient Safety Culture Instrument for Home Care—A Multicentre Cross‐Sectional Study
Source: J Adv Nurs. 2026 Jan 9;82(9):8603–20. doi: 10.1111/jan.70479 (PMC13460978; doi:10.1111/jan.70479)
Supplement: Supplementary file 7 [file JAN-82-8603-s007.docx]

**Appendix S4**

**Item-item and item-total correlations (Spearman)**

**Table S4.**

Item-item and item-total Spearman correlations (r_s_)^a^

| **Dimension/Item** | **Within same dimension (r_s_)** | **With other dimensions’ items (r_s_)** | **Item-total (r_s_)** |
| --- | --- | --- | --- |
| **Teamwork** |  |  |  |
| T1. In this organization, staff treat each other with respect. | 0.33–0.62 | 0.08–0.41 | 0.458 |
| T2. In this organization, staff support one another. | 0.41–0.61 | 0.09–0.41 | 0.462 |
| T3. In this organization, staff feel they are part of a team. | 0.41–0.57 | 0.11–0.46 | 0.539 |
| T4. In this organization, when someone gets really busy, the other team members are ready to help out (e.g., by taking over a visit, a service or other duties). | 0.33-0.41 | 0.06–0.30 | 0.372 |
| T5. In this organization, staff work together as a well-coordinated team. | 0.35-0.62 | 0.13–0.47 | 0.559 |
| **Staffing** |  |  |  |
| S1. In this organization, there is enough staff to handle the workload. | 0.40–0.55 | 0.03–0.38 | 0.484 |
| S2. In this organization, staff generally have to hurry because they have too much work to do. | 0.40–0.53 | 0.09–0.37 | 0.332 |
| S3. In this organization, staff have enough time to respond to clients' needs. | 0.44–0.55 | 0.09–0.45 | 0.578 |
| S4. In this organization, it's hard to keep clients safe because of a high turnover rate. | 0.40–0.44 | 0.14–0.45 | 0.565 |
| **Compliance with procedures** |  |  |  |
| C1. In this organization, staff follow the procedures in place (e.g., procedure in the event of a fall, death, etc.) to care for clients. | 0.29 | –0.01–0.42 | 0.453 |
| C2. In this organization, to get the work done easier, staff often ignore the procedures in place in the organization. | 0.29 | –0.01–0.40 | 0.424 |
| **Training and skills** |  |  |  |
| Z1. In this organization, staff get the training they need to feel confident in the performance of their job. | 0.30–0.48 | 0.20–0.47 | 0.561 |
| Z2. In this organization, staff have enough training on how to handle difficult situations with the clients. | 0.31–0.47 | –0.08–0.37 | 0.453 |
| Z3. In this organization, staff get specific training in client safety. | 0.47–0.48 | –0.10–0.46 | 0.500 |
| **Nonpunitive response to mistakes** |  |  |  |
| R1. In this organization, when staff makes errors, the organization focuses on learning rather than blaming individuals. | 0.25–0.50 | 0.13–0.34 | 0.364 |
| R2. In this organization, staff are afraid to report their mistakes. | 0.27–0.49 | –0.01–0.40 | 0.469 |
| R3. In this organization, staff are treated fairly when they make mistakes. | 0.26–0.55 | 0.16–0.43 | 0.523 |
| R4. In this organization, staff feel safe reporting their mistakes. | 0.23–0.55 | 0.24–0.45 | 0.603 |
| R5. In this organization, staff involved in client safety errors do not receive sufficient support. | 0.24–0.49 | 0.07–0.52 | 0.620 |
| R6.^b^ In this organization, staff members are blamed when a client is harmed. | 0.23-0.27 | –0.10–0.23 | 0.094 |
| **Handoffs and information exchange** |  |  |  |
| H1. Staff have all the information they need to know before taking care of a new client for the first time. | 0.25–0.58 | –0.01–0.38 | 0.560 |
| H2. Staff are notified when there is a change in a client’s care plan/service provision before the visit. | 0.33–0.47 | –0.02–0.38 | 0.458 |
| H3. Staff have all the information they need when clients are transferred from other healthcare facilities (hospitals, nursing homes, etc.). | 0.36–0.58 | –0.02–0.44 | 0.516 |
| H4. Staff have all the information they need to care for clients. | 0.35–0.61 | –0.04–0.44 | 0.506 |
| H5. Staff have all the information they need after clients had a medical consultation (general practitioner, specialist, etc.). | 0.36–0.58 | –0.07–0.48 | 0.517 |
| H6. In this organization, staff have all the information they need when clients are transferred from one team to another within the organization. | 0.34–0.53 | 0.08–0.43 | 0.560 |
| H7.^b^ In this organization, important information is often lost because clients see different staff. | 0.32–0.39 | 0.08–0.44 | 0.528 |
| **Feedback and communication about incidents** |  |  |  |
| F1. In this organization, when staff report something that could harm a client to their manager, someone in the organization takes care of it. | 0.40–0.59 | 0.15–0.51 | 0.650 |
| F2. In this homecare organization, we reflect together about ways to keep incidents from happening again. | 0.43–0.50 | 0.19–0.54 | 0.712 |
| F3. In this organization, staff tell their manager if they see something that might harm a client. | 0.40–0.49 | 0.05–0.49 | 0.467 |
| F4. In this organization, staff discuss ways to ensure clients’ safety. | 0.44–0.56 | –0.01–0.47 | 0.571 |
| F5. In this organization, staff are informed about corrective measures or improvements made based on incident reports. | 0.42–0.56 | –0.03–0.50 | 0.580 |
| **Communication openness** |  |  |  |
| O1. In this organization, staff ideas and suggestions are valued. | 0.25–0.58 | 0.12–0.52 | 0.630 |
| O2. In this organization, it is easy for staff to speak up about problems. | 0.24–0.52 | 0.13–0.47 | 0.583 |
| O3. In this organization, communication is founded on trust. | 0.30–0.58 | 0.16–0.49 | 0.693 |
| O4.^b^ In this organization, staff are afraid to ask questions when something does not seem right. | 0.24–0.30 | –0.02–0.37 | 0.461 |
| **Supervior or manager expectations and actions promoting client safety** |  |  |  |
| P1. My supervisor or manager listens to staff ideas and suggestions about client safety. | 0.66–0.74 | 0.09–0.46 | 0.566 |
| P2. My supervisor or manager expresses satisfaction when work is carried out in compliance with safety procedures. | 0.63–0.66 | 0.13–0.47 | 0.549 |
| P3. My supervisor or manager takes action to address client safety concerns that are brought to their attention. | 0.64–0.80 | 0.10–0.48 | 0.575 |
| P4. My supervisor or manager is actively committed to client safety. | 0.67–0.80 | 0.12–0.50 | 0.651 |
| **Overall perceptions of client safety** |  |  |  |
| B1. Clients are well cared for in this organization. | 0.28–0.65 | 0.10–0.57 | 0.662 |
| B2. This organization does a good job to ensure clients safety. | 0.31–0.69 | 0.07–0.77 | 0.726 |
| B3. This organization provides safe care/services to clients. | 0.26–0.69 | 0.13–0.60 | 0.684 |
| B4. The safety of care/services is not neglected in favour of greater productivity. | 0.22–0.48 | 0.17–0.48 | 0.617 |
| B5.^b^ This organization is doing more for client safety now than it did one year ago. | 0.26–0.31 | –0.01–0.37 | 0.336 |
| **Management support for client safety** |  |  |  |
| M1. The direction listens to staff ideas and suggestions to improve client safety. | 0.46–0.75 | 0.06–0.63 | 0.693 |
| M2. The actions of the management show that client safety is one of the top priorities. | 0.48–0.64 | 0.05–0.69 | 0.707 |
| M3. The direction seems interested in client safety only after an adverse event happens. | 0.41–0.48 | 0.16–0.55 | 0.639 |
| M4. The direction provides adequate resources (e.g., materials, technological resources, human resources, etc.) to improve client safety. | 0.42–0.60 | 0.05–0.64 | 0.644 |
| M5. The direction asks staff how the organization can improve client safety. | 0.41–0.75 | 0.02–0.59 | 0.616 |
| **Organizational learning** |  |  |  |
| L1. In this organization, the same mistakes happen again and again. | 0.48-0.55 | 0.16–0.61 | 0.738 |
| L2. In this organization it is easy to make changes to improve client safety. | 0.48–0.66 | 0.04–0.63 | 0.660 |
| L3. This organization is always doing things to improve client safety. | 0.55–0.66 | 0.06–0.77 | 0.748 |
| L4. When this organization makes changes to improve client safety, it checks to see if the changes worked. | 0.53–0.64 | –0.01–0.64 | 0.674 |
| **Reporting client safety events** |  |  |  |
| A1. When a mistake is caught and corrected before reaching the client, it is reported … | 0.83 | –0.06–0.46 | 0.532 |
| A2. When a mistake reaches the client, but did not harm him, it is reported … | 0.83 | –0.05–0.49 | 0.542 |

*Note.* ^a^ Analyses included all assessed items, even those later removed from the instrument.

Item-total correlations were controlled for overlapping.

^b^ These items were removed from the final version of the instrument

Negative correlations correspond to items R6 and B5.

This is an unofficial, not validated translation of the items of the French version of the instrument.
